# Lactoferrin Modulates Intestinal Morphology and Microbiota Homeostasis in Chronic Social Defeat Stress Mice

**DOI:** 10.3390/molecules31203614

**Published:** 2026-10-10

**Authors:** Karolina Orzeł-Gajowik, Mariusz Popek, Zuzanna Sulej, Marta Obara-Michlewska, Paweł Kowalczyk, Iwona Bukowska-Ośko, Karol Perlejewski, Karol Kramkowski, Agnieszka Pollak, Magdalena Zielińska

**Affiliations:** 1 Department of Neurotoxicology, Mossakowski Medical Research Institute, Polish Academy of Sciences, 5 Pawińskiego Str., 02-106 Warsaw, Poland; mpopek@imdik.pan.pl (M.P.); zsulej@imdik.pan.pl (Z.S.); mobara@imdik.pan.pl (M.O.-M.); 2 Doctoral School of Translational Medicine, Centre of Postgraduate Medical Education, Marymoncka 99/103, 01-813 Warsaw, Poland; 3 Department of Animal Nutrition, The Kielanowski Institute of Animal Physiology and Nutrition, Polish Academy of Sciences, Instytucka 3, 05-110 Jabłonna, Poland; p.kowalczyk@ifzz.pl; 4 Department of Immunopathology, Infectious and Parasitic Diseases, Medical University of Warsaw, Pawińskiego 3c, 02-106 Warsaw, Poland; iwona.bukowska@wum.edu.pl (I.B.-O.); karol.perlejewski@wum.edu.pl (K.P.); 5 Department of Physical Chemistry, Medical University of Białystok, Kilińskiego 1, 15-089 Białystok, Poland; kkramk@wp.pl; 6 Department of Medical Genetics, Medical University of Warsaw, Pawińskiego 3c, 02-106 Warsaw, Poland; agnieszka.pollak@wum.edu.pl

**Keywords:** lactoferrin, chronic social defeat stress, gut–brain axis, microbiota, intestinal morphology, anxiety

## Abstract

Chronic social stress is increasingly linked to gut–brain axis dysfunction, yet gut-targeted interventions remain underexplored. This study investigated whether oral lactoferrin (L) administration modulates the intestinal and behavioral consequences of chronic social defeat stress (CSDS) in male C57BL/6J mice. Animals were randomly assigned to four groups—Control, Control+L, CSDS, and CSDS+L—and received lactoferrin (5 mg/kg/day) or vehicle via oral gavage over 15 days concurrent with the CSDS paradigm. Behavioral assessments (fur condition scoring, splash test, open field test), intestinal permeability (FITC-dextran assay), whole gut transit, small-intestine morphology and tight junction protein expression (ZO-1, claudin-5), gut microbiota composition (16S rRNA gene sequencing), and oxidative DNA damage repair activity were evaluated. CSDS induced significant fur deterioration, body weight loss, and reduced grooming behavior, all of which were partially attenuated by L supplementation. In the ileum, CSDS increased crypt number and trended toward decreased claudin-5 expression, whereas L treatment increased crypt depth relative to stressed controls. Although alpha and beta diversity remained unchanged, CSDS prevented the physiological decline of the Firmicutes-to-Bacteroidetes ratio observed in controls, was associated with differences in gut microbiota composition, and L partially restored this trajectory. DNA repair enzyme activities were unaffected. These findings indicate that L mitigates stress-induced somatic deterioration and intestinal alterations, positioning it as a promising gut-targeted nutritional adjunct for stress-related disorders.

## 1. Introduction

Anxiety, contributing to depressive disorders, affects hundreds of millions of people across all age groups and is among the leading causes of disability worldwide [1]. Currently, pharmacotherapy is the leading treatment for these diseases; however, at least 50% of patients respond poorly [2]. Furthermore, conventional treatments are often associated with adverse effects, including dependency and metabolic disturbances [3]. These limitations underscore the need to elucidate the peripheral biological underpinnings of stress-related diseases and to identify adjunctive interventions targeting systemic mechanisms.

The pathogenesis of depression is increasingly linked to organismal dishomeostasis [4,5]. Systemic and central dysfunctions involve systemic inflammation stemming from gut dysbiosis and compromised intestinal barrier integrity [6,7]. External stressors, particularly social stress, can disrupt epithelial tight junction (Tj) composing proteins, increase intestinal permeability, and facilitate translocation of Gram-negative bacteria that produce lipopolysaccharide (LPS) released into the circulation, thereby triggering an increase in pro-inflammatory cytokines [8]. In parallel, microbial signaling metabolites such as short-chain fatty acids (SCFA), tryptophan derivatives, and indoles modulate immune responses, and intestinal and blood–brain barrier permeability, affecting central neuroinflammation and/or neurotransmission, and are in a concerted way implicated in the pathogenesis of anxiety and depression [9,10]. Clinical studies revealed strain differences and composition diversity of the gut microbiota in patients with anxiety [11] and transplantation of the gut microbiota from anxious individuals to germ-free mice, which transfers anxiety-like phenotypes [12,13,14]. Thus, microbiome control or modulation represents a promising therapeutic avenue.

Lactoferrin (L) is a naturally produced, multifunctional, iron-binding, 80 kDa glycoprotein found in exocrine secretions (e.g., tears, saliva) and is particularly abundant in mammalian milk [15]. In the intestine, L modulates microbial ecology by sequestering iron from pathogens, providing glycans and iron to commensals, and modulating the proliferation and composition of resident microflora [16]. L has also been shown to enhance mucosal barrier function, support epithelial regeneration, and modulate adaptive and innate immune responses in the gut [17,18,19,20,21,22]. Previous reports indicate that L can ameliorate metabolic and inflammatory disturbances in obesity, type 2 diabetes, and stress, partly via microbiota-dependent mechanisms [23,24,25,26]. These pleiotropic properties make L an attractive candidate for targeting stress-induced dysregulation.

In this study, we hypothesized that chronic social defeat stress (CSDS) would induce alterations in behavioral, somatic, intestinal, and microbiota-related parameters, and that lactoferrin (L) ingestion would modulate these stress-associated changes. CSDS exposure and lactoferrin administration were examined as two experimental factors, each with two levels (absent/present). Using a 15-day CSDS paradigm, we assessed: (i) anxiety-like and self-care-related behaviors (fur condition, splash test, open field test); (ii) systemic biochemical markers; (iii) intestinal permeability and motility; (iv) small-intestinal morphology and tight junction protein expression; (v) gut microbiota composition via 16S rRNA gene sequencing; and (vi) oxidative DNA damage repair activity in intestinal tissues.

## 2. Results and Discussion

### 2.1. Stress-Induced Somatic Deterioration

**Figure 1:**
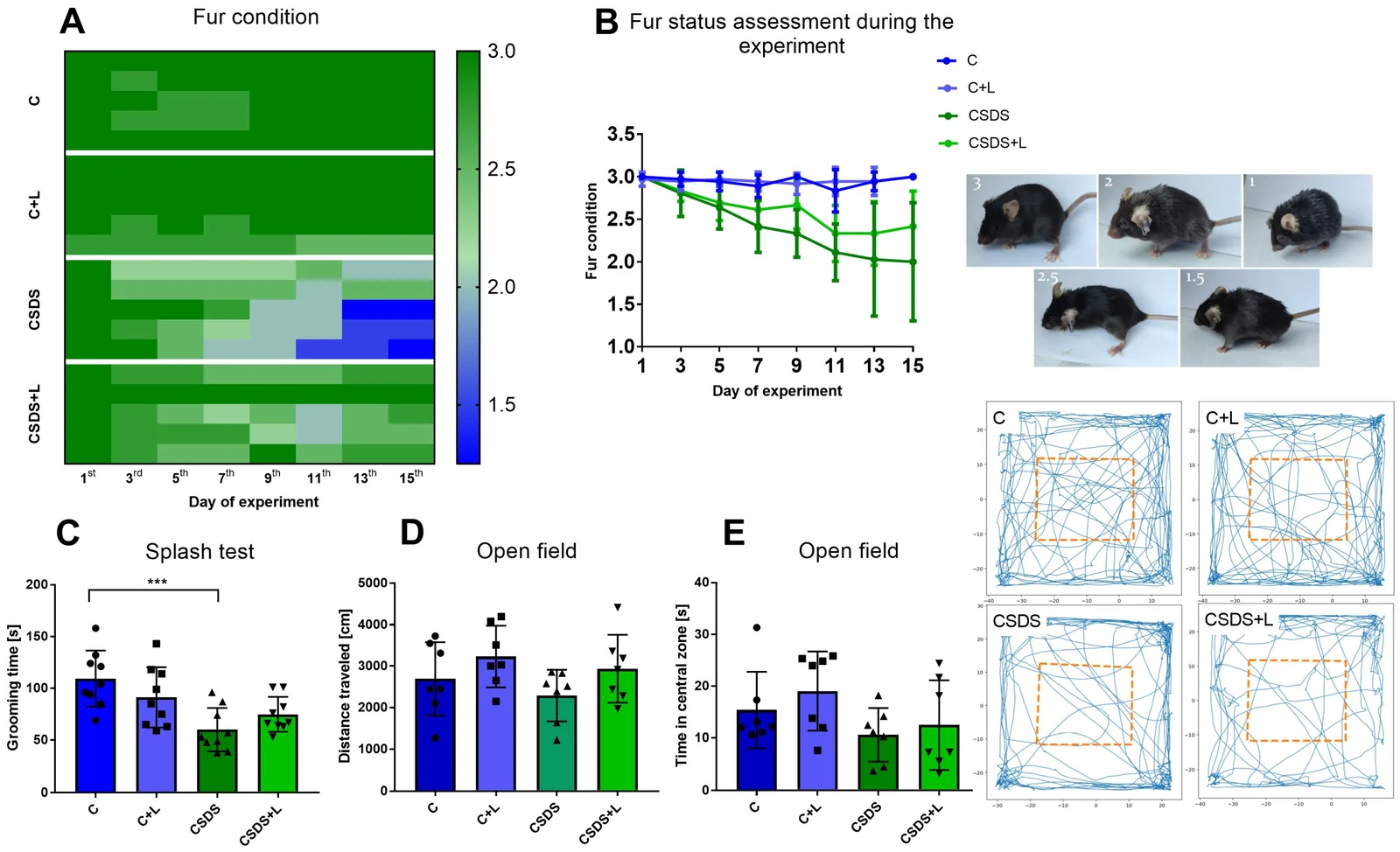
Physical and behavioral condition assessment in mice exposed to chronic social defeat stress (CSDS) and lactoferrin (L) administration. (**A**) Heatmap of mice fur status during the experiment. (**B**) Fur condition assessment scores of mice during the experiment. (**C**) Grooming time during the Splash Test for individual mice. (**D**) Distance traveled in the open field test. (**E**) Central zone time in the open field test with representative traces of individual mouse locomotor activity. The results are the mean ± SD. *n* = 9 for the Splash Test and for the fur condition; *n* = 7 for open field; *** *p* ≤ 0.001 vs. control (C).

**Figure 2:**
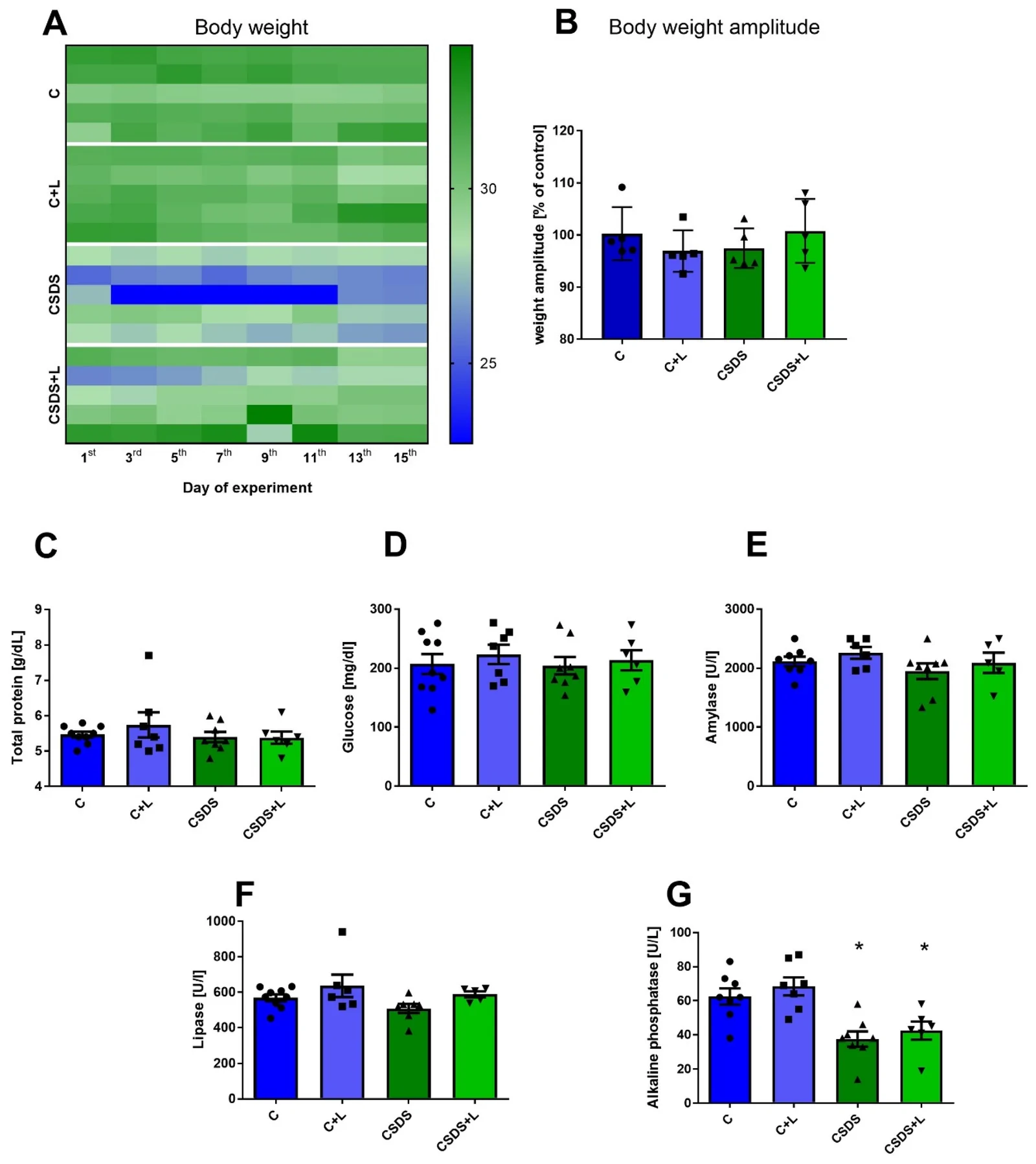
Effect of chronic social defeat stress (CSDS) and lactoferrin (L) administration on body weight and biochemical parameters. (**A**) Heatmap showing body weight changes during the experiment. The color gradient represents body weight in grams. (**B**) Bar graph of body weight amplitude (percent of control) across groups. (**C**) Total protein concentration (g/dl). (**D**) Glucose levels (mg/dl). (**E**) Amylase activity (u/l). (**F**) Lipase activity (u/l). (**G**) Alkaline phosphatase activity (u/l). Results are presented with individual data points and mean ± SD across groups. *n* = 5 for body weight analysis and *n* = 6–9 for biochemical analysis in serum. * *p* < 0.05 vs. control (C).

CSDS exposure induced a progressive decline in physical condition (Figure 1 and Figure 2). Control groups (C and C+L) maintained stable fur scores (~3.0) and body weight. Mice in the control group and those treated with L had body weights consistently averaging 30 g. Conversely, the CSDS group demonstrated a decrease in body weight throughout the experiment, with weights falling below 25 g by the tenth day. The cohort subjected to CSDS and administered L exhibited a partial recovery, with body weight stabilizing at around 27 g.

Body weight amplitude (Figure 2B), measured as a percentage of the control, showed a tendency to decrease in the CSDS group. The CSDS+L group presented an increase in weight amplitude compared to the untreated CSDS group. However, neither group fully recovered to control levels.

In contrast, CSDS mice exhibited significant fur deterioration starting from Day 7 (score ~2.0 by Day 15; Figure 1A,B) and substantial weight loss (Figure 2A,B). Lactoferrin treatment (CSDS+L) partially attenuated these effects, resulting in improved fur scores and reduced weight loss compared to the untreated CSDS group, although values did not fully return to control levels.

### 2.2. Self-Care and Anxiety-like Behavior of Animals

The splash test was used to analyze the grooming behavior of mice. In the splash test, CSDS mice showed a significant reduction in grooming time compared to controls (*p* < 0.01), indicative of depressive-like neglect. L administration tended to increase grooming time in stressed mice (CSDS+L), suggesting restored motivational behavior (Figure 1C). In the open field test, no significant differences were observed between groups regarding total distance traveled (Figure 1D) or time spent in the central zone (Figure 1E). This suggests that while CSDS affected somatic and self-care measures, it did not induce overt anxiety-like locomotor inhibition in this specific cohort, nor did L affect these parameters.

The CSDS procedure was selected for its well-established translational relevance to human psychosocial stress and its ability to recapitulate core features of anxiety-related phenotypes, including reduced self-care and motivational deficits [27]. Importantly, the CSDS protocol would require modification for females due to the lack of consistent ethologically relevant aggression, as described by Harris et al. (2018) [28]. The observed impairments in grooming behavior and fur condition are widely accepted preclinical indicators of anhedonia and behavioral withdrawal, paralleling clinical symptoms observed in anxiety and depressive disorders. The ability of L to improve these metrics suggests a systemic benefit, potentially mediated through improved nutritional status (evidenced by weight maintenance) or stabilization of peripheral homeostasis. Interestingly, we did not observe significant differences in anxiety-like behavior in the open field test. This dissociation between somatic/stress-like symptoms and anxiety-like locomotion is not uncommon in CSDS models and may depend on the specific strain or stress duration. In our study, all mice exposed to CSDS were included in the analysis. This approach may have contributed to the attenuation of behavioral differences. Notably, previous work has demonstrated that focusing specifically on susceptible animals can enhance the detection of stress-induced behavioral and microbiome alterations; inclusion of resilient individuals may mask or dilute these effects [29]. The somatic and behavioral changes observed in CSDS mice align with clinical manifestations of human stress-related disorders. Deterioration of fur condition and reduced grooming behavior in the splash test are objective preclinical markers of anhedonia and reduced self-care, which parallel the neglect of self-maintenance routines, loss of interest in daily activities, and psychomotor retardation commonly reported by patients with depressive disorder. Similarly, CSDS-induced body weight loss reflects the appetite and metabolic disturbances that affect up to 60% of individuals with depression [30].

### 2.3. Biochemical and Physiological Parameters Determination

Plasma biochemical analysis showed no significant differences in total protein, glucose, amylase, or lipase activity across groups. However, alkaline phosphatase (ALP) levels were significantly lower in the CSDS group compared to controls (*p* < 0.05), with L treatment showing a non-significant trend toward restoration (Figure 2G).

### 2.4. Intestinal Permeability and Motility Examination

**Figure 3:**
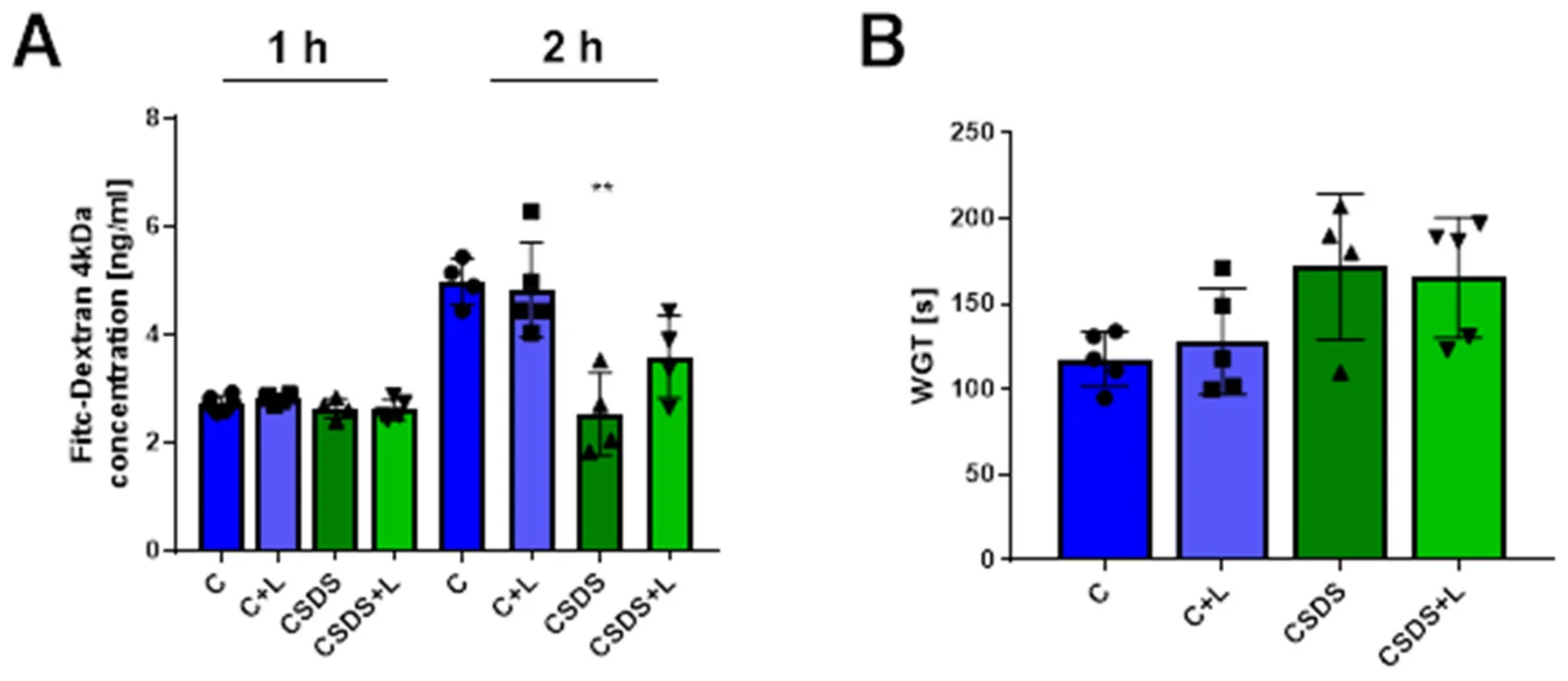
Effect of chronic social defeat stress (CSDS) and lactoferrin (L) administration on intestinal permeability and whole gut transit (WGT). (**A**) Bar graphs of FITC-Dextran (4 kDa) concentration (ng/mL) at two time points: 1 h (**left**) and 2 h (**right**) post-administration. (**B**) Bar graph of WGT across groups. The results are the mean ± SD; *n* = 4–5; ** *p* < 0.01 vs. control.

Plasma FITC-dextran appearance was used as an indirect measure related to intestinal permeability (Figure 3A). At the 1 h time point, no significant differences in FITC-dextran concentration were observed between groups, with all experimental conditions showing similar levels around 3 ng/mL. At the 2 h time point, mice exposed to CSDS exhibited a lower plasma FITC-dextran concentration compared to controls. Treatment with L was associated with a partial increase in plasma FITC-dextran concentration at 2 h, although levels did not fully return to those of the control group. The reduced appearance of FITC-dextran in plasma may therefore reflect delayed gastric emptying or intestinal transit rather than enhanced barrier integrity.

Whole Gut Transit (WGT) assessment revealed a trend toward delayed motility in the CSDS group (longest transit time), which was partially normalized in the CSDS+L group (Figure 3B). Thus, FITC-dextran levels may reflect gastrointestinal transit rather than intestinal barrier function. Notably, disruptions in gut homeostasis, the focus of L intervention in this study, are increasingly recognized as a key contributor to human mood disorders [31]. Clinical studies have demonstrated that patients with depression and anxiety are significantly more likely to report gastrointestinal symptoms and exhibit alterations in gut microbiota composition, reduced gut barrier integrity, and altered intestinal transit times—all endpoints we assessed in this study. By demonstrating that L can attenuate some of these stress-induced intestinal and behavioral changes in CSDS mice, our findings provide preclinical data exploring L as an adjunctive strategy to target gut–brain peripheral network dysfunction in humans exposed to chronic psychosocial stress or suffering from mood disorders.

### 2.5. Intestinal Morphology and Tight Junction Proteins

**Figure 4:**
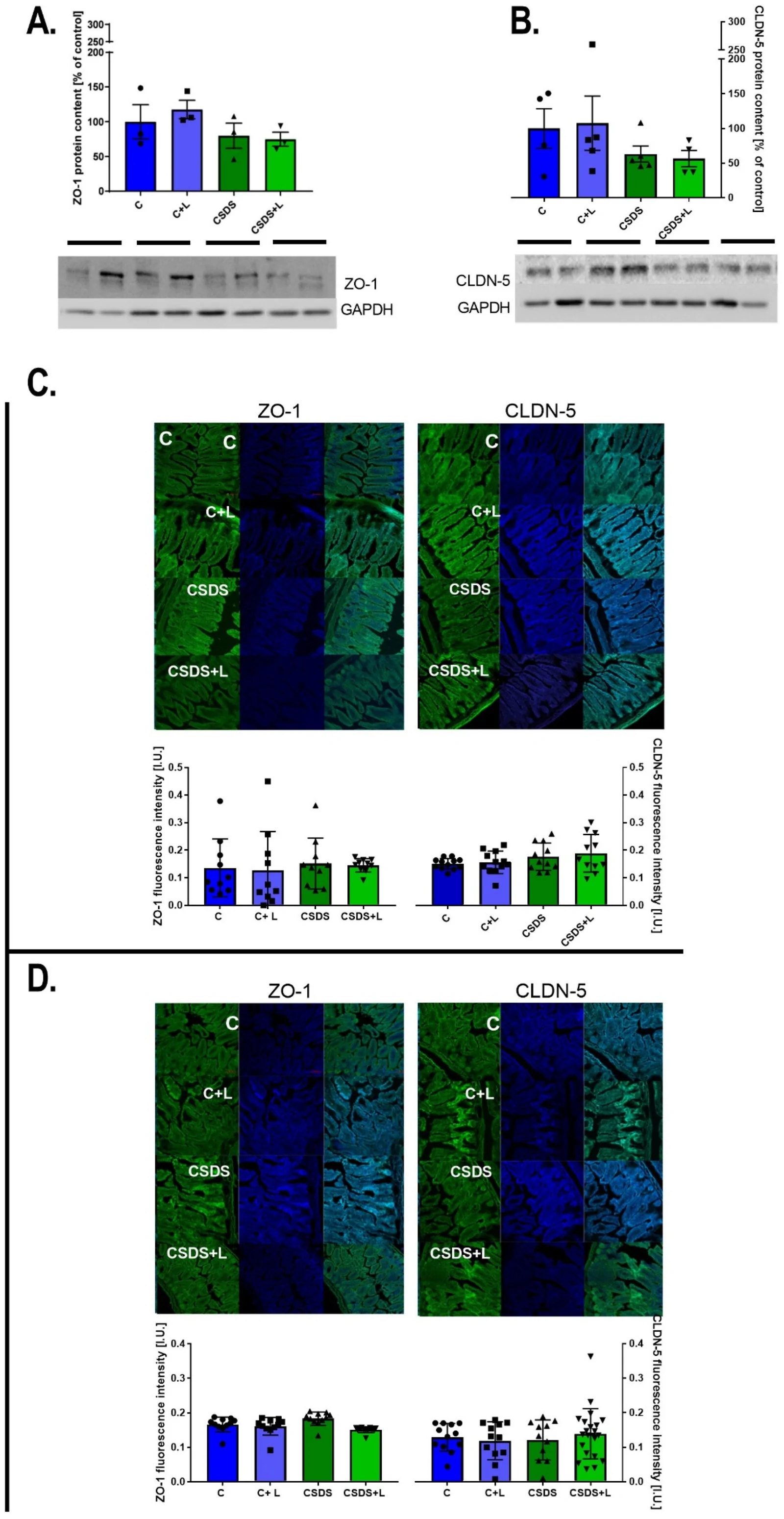
Effect of chronic social defeat stress (CSDS) and lactoferrin (L) administration on zona occludens 1 (ZO-1) and claudin-5 (CLDN-5) protein expression in intestinal tissue (jejunum vs. ileum). Bar graph of ZO-1 (**A**) and CLDN-5 (**B**) protein content (percent of control) with representative Western blot images (lower panels). Bar graph of ZO-1 and CLDN-5 fluorescence intensity in jejunum (**C**) and ileum (**D**) intestinal tissue with representative images (upper panels). The results are the mean ± SD with individual data points; *n* = 3 for ZO-1 protein content and IHC analysis, *n* = 4–5 for CLDN-5 protein content analysis.

Western blot analysis revealed that ZO-1 protein expression tended to be lower in the CSDS group compared with controls (approximately 10% lower, Figure 4A), although this difference did not reach statistical significance. Treatment with L did not restore ZO-1 levels.

Western blot analysis for claudin-5 (Figure 4B) followed a similar pattern. The CSDS group demonstrated a modest, although not statistically significant, decrease in claudin-5 expression by ~40% of control values, while the CSDS+L group exhibited no changes in claudin-5 protein levels compared to CSDS.

**Figure 5:**
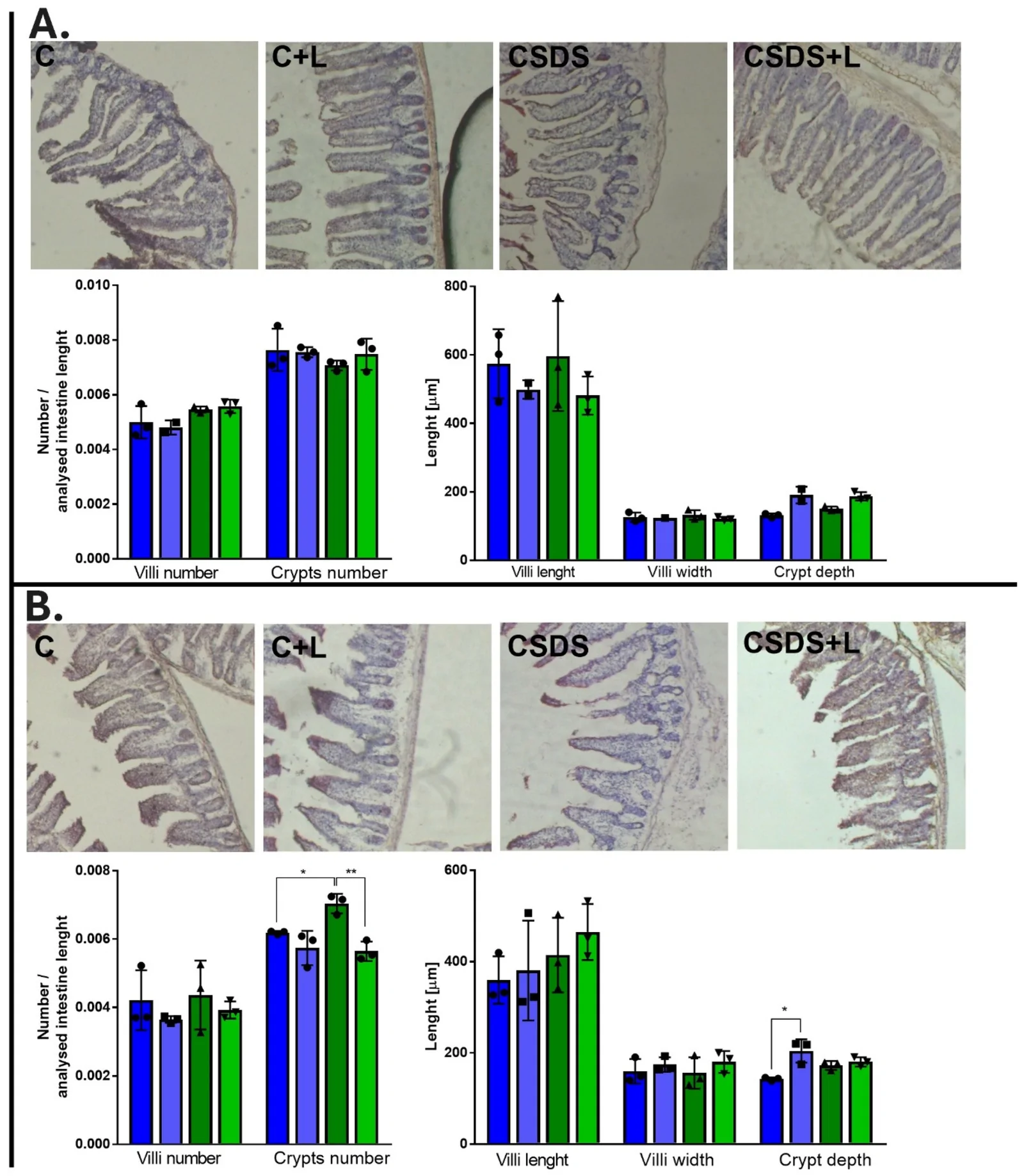
Effects of chronic social defeat stress (CSDS) and lactoferrin (L) administration on intestinal morphology in the jejunum and ileum. Bar graphs with quantitative data of villi and crypts number, as well as villi length, width, and crypt depth in the jejunum (**A**, lower panel) and ileum (**B**, lower panel). Representative tissue images are presented in upper panels. Results are the mean ± SD; *n* = 3 (4–7 intestinal sections per animal analyzed independently for each segment); * *p* < 0.05, ** *p* < 0.01 for the comparisons indicated by brackets.

Immunofluorescence intensity (Figure 4C,D showed no alterations in treated groups. CSDS induced region-specific morphological changes. The intestinal morphology of the jejunum and ileum was analyzed across four experimental groups. Representative microscopic images of jejunal and ileal sections show typical villus and crypt structures (Figure 5, upper panels).

In the jejunum, quantitative analysis revealed no significant differences in villi and crypt numbers nor in villi length, width, or crypt depth across the groups (Figure 5A).

In the ileum, differences in morphology are more pronounced. CSDS significantly increased crypt number compared with controls, whereas L treatment reduced crypt number in CSDS+L mice compared with the CSDS group, restoring it to approximately control levels (Figure 5B). L significantly increased crypt depth in non-stressed mice (C+L vs. C), whereas CSDS did not significantly affect this parameter (Figure 5B).

These morphological changes suggest that chronic stress and L treatment conditions exert selective effects on small intestine architecture, particularly in the ileum, possibly reflecting adaptive responses or structural remodeling in response to stress and intervention.

The partial restoration of ileal crypt morphology/number and the trend toward normalized nutrient absorption (indicated by biochemical markers) support the hypothesis that L acts as a gut protector.

Intestinal barrier integrity elements, claudin-5 and ZO-1, which regulate paracellular permeability, are implicated in various gastrointestinal diseases where barrier dysfunction occurs [32]. Tj proteins uniquely contribute to the selective permeability of the intestinal epithelium, balancing nutrient absorption and protection against harmful agents. Although reduced plasma FITC-dextran levels were observed in CSDS mice, this finding should not be interpreted as evidence of improved intestinal barrier integrity. Plasma FITC-dextran appearance provides an indirect assessment related to intestinal permeability and may be influenced by gastrointestinal transit and luminal delivery of the tracer. Several non-exclusive mechanisms may therefore account for the observed differences, including delayed intestinal transit, altered mesenteric blood flow, or other factors affecting the systemic appearance of the tracer. Thus, in the present study, the lower plasma FITC-dextran concentration cannot be interpreted as evidence of a tighter intestinal barrier in isolation. These limitations highlight the need for complementary permeability assessments using size-selective probes, bacterial translocation assays, and ex vivo Ussing chamber analyses.

### 2.6. Microbiome Analysis

#### 2.6.1. Diversity Metrics

**Figure 6:**
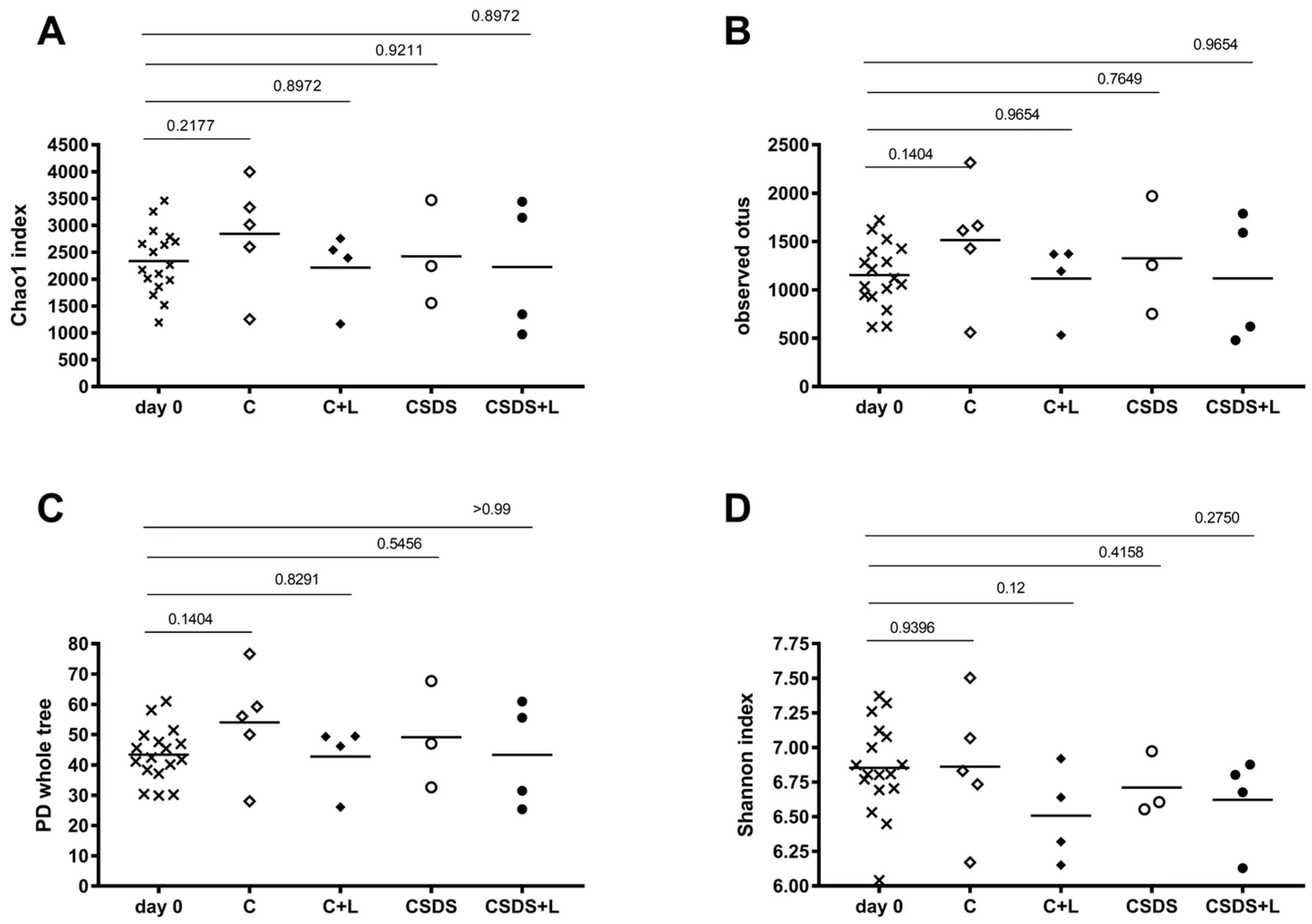
The effect of chronic social defeat stress (CSDS) and lactoferrin (L) administration on alpha diversity measures of the gut microbiota from animals. (**A**) Chao1 index. (**B**) Operational taxonomic units (OTUs). (**C**) Phylogenetic diversity (PD) whole tree. (**D**) Shannon index. *n* = 3–4.

Alpha diversity analysis indices: Chao’s Richness Estimator (Chao 1) index, observed number of operational taxonomic units (OTUs), phylogenetic diversity (PD) whole tree, and Shannon index revealed no statistically significant differences between the CSDS and C group after 15 days of the experiment (Figure 6). Moreover, there was no significant difference in the alpha diversity between mice receiving L and those not supplemented with L in both the C and the CSDS groups of animals (*p* > 0.05).

Statistical differences (*p* < 0.05) between alpha diversity measures in groups examined by the Mann–Whitney two-tailed test were not observed. Similarly, no statistically significant differences in beta diversity distances (weighted UniFrac) of the intestinal microbiome were observed among the CSDS and C groups at 0 and 15 days of the experiment or between supplemented and non-supplemented L groups of animals.

#### 2.6.2. Microbiota Compositional Changes

**Figure 7:**
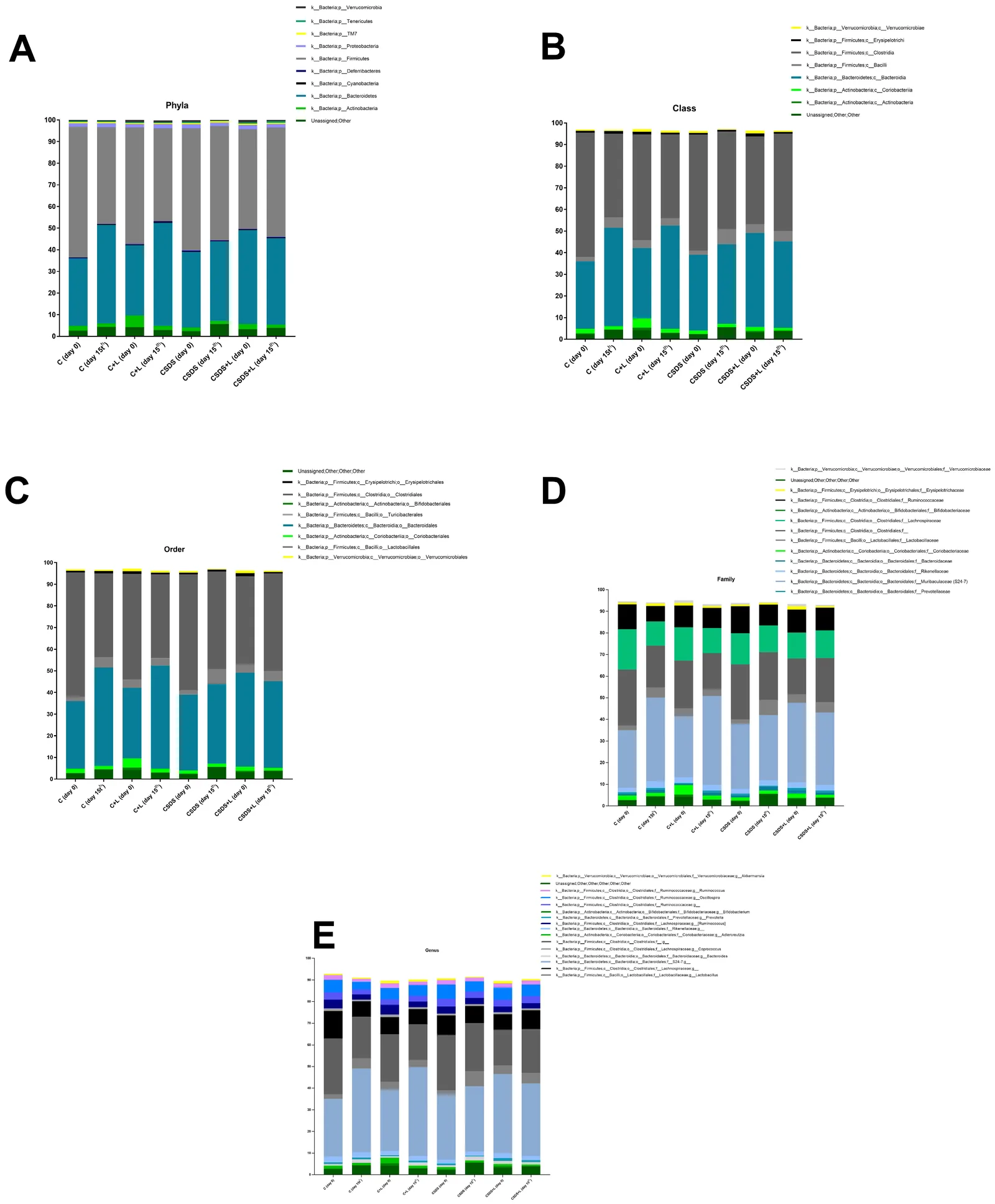
Relative abundance above 1% at different bacterial taxonomic levels in individuals with chronic social defeat stress (CSDS), control (C), and with lactoferrin supplementation (L); (**A**) Relative abundance of bacterial phyla; (**B**) Relative abundance of bacterial classes; (**C**) Relative abundance of bacterial orders; (**D**) Relative abundance of bacterial families; (**E**) relative abundance of bacterial genera. *n* = 3–4.

Based on the 16S sequencing, the results were classified into 9 bacterial phyla. Five phyla, *Bacteroidetes*, *Firmicutes*, *Actinobacteria*, *Proteobacteria*, and *Verrucomicrobia*, showed relative abundance exceeding the filtering criteria (>1% in at least one study group) (Figure 7). A shift in the dominant bacterial phyla, with Bacteroidetes becoming the most abundant, followed by *Firmicutes*, was observed at the 15-day mark of the experiment in the control group (35.5% vs. 45.5% of *Bacteroidetes* and 54.2% vs. 44.7% of *Firmicutes* at 0 day vs. 15 day, respectively) and the C+L group (35.5% vs. 47.7% of *Bacteroidetes* and 54.2% vs. 42.9% of *Firmicutes* at 0 day vs. 15 day, respectively).

The relative abundance of these two phyla in the depressed subgroup showed no changes during 15 days of experiment (35.5% vs. 36.8% of *Bacteroidetes* and 54.2% vs. 52.9% of *Firmicutes* at 0 day vs. 15 day, respectively) and some depletion of *Firmicutes* and elevation of *Bacteroidetes* in the L subgroup (35.5% vs. 40% of Bacteroidetes and 54.2% vs. 50.6% of *Firmicutes* at 0 day vs. 15 day, respectively). The relative abundance of the *Actinobacteria* phylum showed depletion at 15 days of observation (Figure 7).

**Figure 8:**
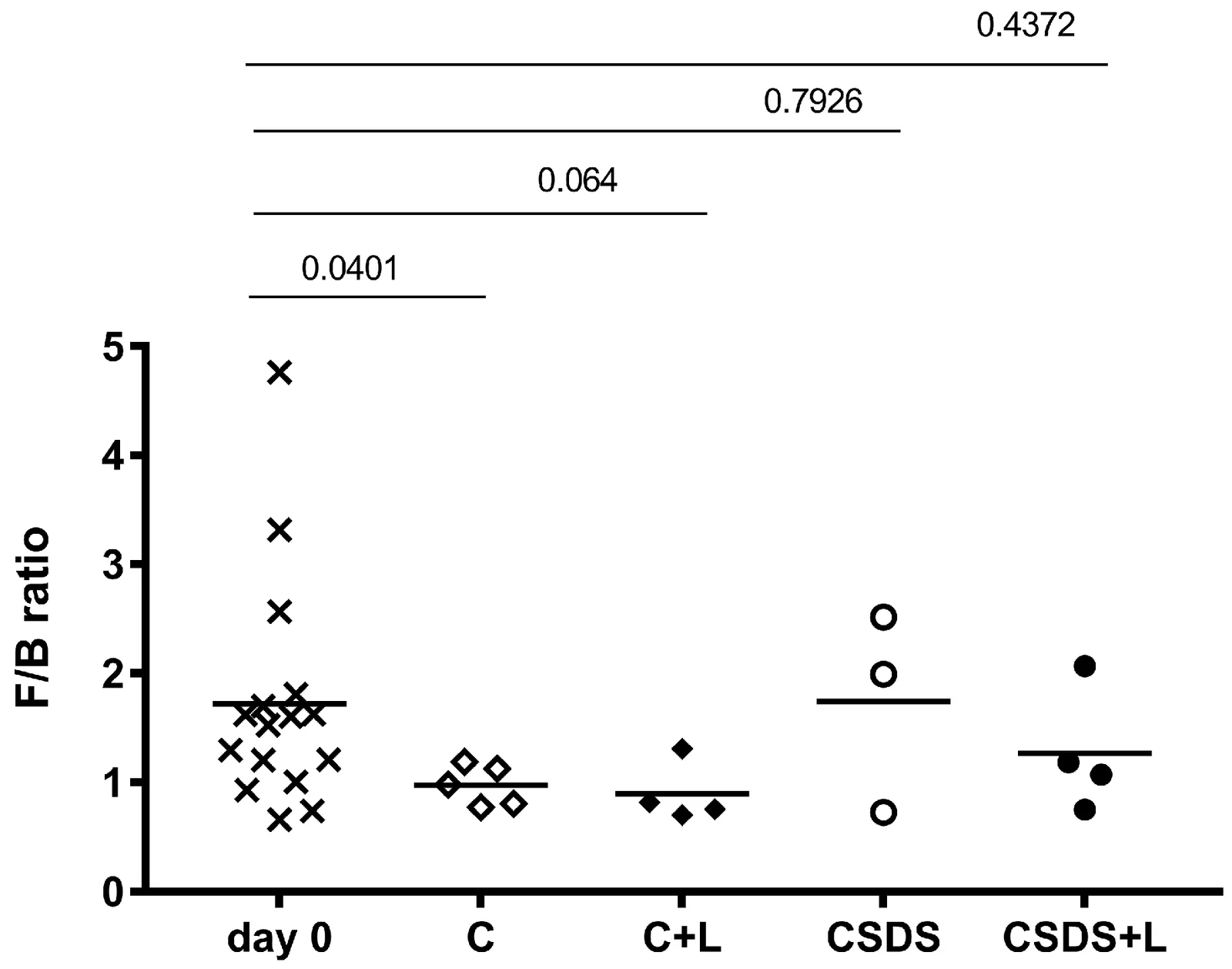
*Firmicutes/Bacteroidetes* (F/B) ratio of control (C) mice, mice with chronic social defeat stress (CSDS), and mice with lactoferrin (L) supplementation. Statistical differences between groups were examined by the Mann–Whitney two-tailed test (*n* = 3–5).

The *Firmicutes-to-Bacteroidetes* (F/B) ratio was significantly lower at the end of the experiment in the control group (*p* = 0.0401) and was unaltered in the control group treated with L (*p* = 0.064). There were no differences in the F/B ratio between other analyzed groups (Figure 8).

None of the observed changes in gut microbiota composition had reached statistical significance; however, some trends were observed. At the class level, the *Bacteroidia* (order *Bacteroidales*) and *Clostridia* (order *Clostridiales*) predominated in all subgroups. However, the relative abundance of these two classes varies between 0 and 15 days of the experiment—*Bacteroidia*: 35.5% vs. 45.5% of the C subgroup and 47.7% of the C+L subgroup; *Clostridia*: 50.1% vs. 38.7% of the C subgroup, 38.7% of the C+L subgroup, 45% of the CSDS subgroup, and 45% of the CSDS+L subgroup, respectively (Figure 7). The third most abundant bacterial class in all subgroups was *Bacilli* (order *Lactobacillales*), demonstrating higher prevalence after 15 days of the experiment in the C and CSDS subgroups (2.95% vs. 4.9% and 7.1%—0 days vs. 15 days, respectively). The *Actinobacteria* phyla were assigned to two classes: *Actinobacteria* and *Coriobacteria*, represented by *Bifidobacteriales* and *Coriobacteriales* orders, respectively. The *Coriobacteriales* order was represented in different proportions between 0 and 15 days in C+L and C subgroups: 2.48% vs. 1.5% and 4.3% vs. 1.8%.

At the family level, *Muribaculaceae* (formerly S24-7) emerged as dominant, accounting for 30.43% and enriched for 15 days to 38.6% in the C subgroup, to 41.2% in the C+L subgroup, and to 33.5% in the CSDS+L subgroup (Figure 8). Only the CSDS group presents unchanged *Muribaculaceae* abundance (30.1%). Additionally, an increase in *Lactobacillaceae,* with the genus *Lactobacillus*, was found in the C-treated or not with L mouse groups (from 2.93% to 4.7% in C and 3.2% in C+L) and in CSDS groups treated or not with L (7.1% in CSDS and 4.8% in CSDS+L). In contrast, the gut microbiota presented abundance depletion in the C and CSDS groups at day 0 as compared to day 15 of the experiment of *Bacteroidaceae*, represented by the genus *Bacteroides* (2.78% vs. 1.6% in C, 1.6% in C+L, 1.8% in CSDS and 1.1% in CSDS+L, respectively), as well as *Lachnospiraceae* (15.13% vs. 11.2% in C; 11.6% in C+L 12.3% in CSDS; and 12.8% in CSD+L, respectively), and *Ruminococcaceae* (11.15% vs. 7.2% in C; 9.4% in C+L; 9.6%; 10.5% in CSDS and CSDS+L, respectively) represented by the genera *Oscillospira* and *Ruminococcus* (Figure 8). A decrease in the *Oscillospira* genus was observed in the C and CSDS groups (5.8% vs. 3.6% and 4.8%, respectively). PICRUSt-based KEGG pathway analysis showed no significant differences in predicted metabolic pathways between the experimental groups.

In contrast to studies that reported profound dysbiosis in stress-related models [33,34], our 16S rRNA analysis revealed that CSDS did not induce drastic alterations in alpha or beta diversity. While L appears to stabilize the abundance of genera such as *Oscillospira* and *Lactobacillus*, we cannot conclude that L exerts a modulatory effect on microbiome composition in this specific paradigm. The data presented in this study provided evidence that L did not significantly change the microbiome abundance under stress conditions. However, we observed stabilization of some bacterial shifts in the CSDS and control groups, including prevention of the *Lactobacillus* genus increase as well as the *Oscillospira* genus and *Bacteroidales* order decrease. This observation is consistent with a beneficial effect on body wellness, since the *Oscillospira* genus is considered an anti-inflammatory bacterium, and *Bacteroidales* is positively associated with leanness and health [35].

*Lactobacillus* abundance has been associated with reduced neuroinflammation, lower circulating corticosterone, and enhanced hippocampal neurogenesis in several preclinical models [36,37]. However, conflicting evidence indicates that specific *Lactobacillus* strains or dosing paradigms may induce stress-like phenotypes and social deficits under certain experimental conditions [36,38,39]. These discrepancies highlight the strain-specific and context-dependent nature of *Lactobacillus*–host interactions, underscoring the need for cautious interpretation of genus-level changes. The results showed that the increase in the *Lactobacillus* genus is inversely correlated with the most abundant *Muribaculaceae* strain. The *Lactobacillus* genus is primarily a lactic acid producer that causes the environment to acidify and, in turn, inhibits the growth of the *Muribaculaceae*. However, no significant changes in *Muribaculaceae* were found in studies of chronic unpredictable mild stress (CUMS) and rodent models of depression [40].

In our study, however, at the family level, a trend toward increased relative abundance of *Muribaculaceae* over the 15-day experimental period was observed in C, C+L, and CSDS+L mice, whereas *Muribaculaceae* abundance remained unchanged in the CSDS group.

Although these differences did not reach statistical significance, the absence of *Muribaculaceae* expansion in CSDS mice contrasted with the upward trajectory observed in control animals, suggesting that chronic psychosocial stress may alter the normal temporal remodeling of this metabolically relevant bacterial family.

*Muribaculaceae* are considered commensals involved in polysaccharide fermentation and SCFA-associated metabolic networks; therefore, their time-dependent enrichment observed in control animals may reflect temporal adaptation of the gut microbiome during the experimental period [41].

Recent experimental evidence suggests that enrichment of *Muribaculaceae* may exert anti-inflammatory and barrier-protective effects [42].

Further, our findings appear to align with previous observations by Donovan and colleagues who demonstrated in a murine model of chronic social stress that stress-induced behavioral disturbances were accompanied by shifts in gut microbial taxa, including members of the *Muribaculaceae* family, supporting the notion that psychosocial stress may affect not only microbiota composition per se but also the ecological dynamics of metabolically relevant commensals [43].

Unlike *Muribaculaceae*, whose enrichment was absent in CSDS mice, *Lactobacillus* showed the highest relative abundance in the CSDS group, suggesting that these taxa followed divergent trajectories under chronic psychosocial stress.

The increased abundance of *Lactobacillus* in stress-exposed mice may represent a stress-associated microbial response, as members of this genus have been implicated in host stress resilience, neuroimmune modulation, and maintenance of gut homeostasis under adverse conditions.

Interestingly, although *Lactobacillus* reached the highest relative abundance in CSDS mice, this increase was attenuated by L supplementation. This may suggest that, rather than suppressing a beneficial microbial response, lactoferrin reduced the need for stress-induced compensatory expansion of Lactobacillus by partially restoring microbial homeostasis [44].

*Bacteroidetes* and *Firmicutes* were the most abundant bacteria in the four groups studied here. The *Firmicutes*-to-*Bacteroidetes* ratio (F/B) has frequently been used as a coarse indicator of gut microbiota composition and dysbiosis. Although previous studies have suggested that alterations in the F/B ratio reflect pathology-associated changes in gut microbiota homeostasis [45], the direction and biological interpretation of these changes remain debated [46]. Alterations in this ratio have also been associated with stress exposure and may contribute to stress-related inflammatory responses, although its interpretative value remains context-dependent [46,47].

The F/B ratio decreased significantly in control mice over the 15 days (*p* = 0.0401), whereas no significant change was observed in the C+L group (*p* = 0.064). As the F/B ratio is a descriptive compositional metric reflecting the relative abundance of two major bacterial phyla, changes in this ratio should be interpreted cautiously. In the present study, the observed temporal changes do not by themselves provide evidence of microbiome maturation, improved microbiome health, or ecological adaptation [35]. Since the F/B ratio decreased in both the C and C+L groups, this shift may reflect a time-dependent adaptive change in microbiota composition rather than an effect of externally imposed dietary or husbandry changes. This reduction may represent a physiological trajectory of microbiome maturation and ecological adaptation. Since the gut microbiota is increasingly understood as a dynamic and adaptive ecosystem rather than a static entity, temporal changes in microbiota composition, rather than absolute values, may more accurately reflect physiological adaptation [48].

In CSDS mice, the F/B ratio remained relatively stable over the experimental period. This pattern indicates a different temporal trajectory in the relative abundance of Firmicutes and Bacteroidetes compared with control animals; however, the biological significance of this difference cannot be determined from the F/B ratio alone. L administration was associated with a trend toward a lower F/B ratio in stressed mice, although this change did not reach statistical significance. The biological significance of this change remains uncertain.

In line with this, Hu et al. [46] found that *Bacteroides* were significantly enriched in moderately and severely stressed animals, while there was no change in the mild stress group. These findings suggested that the gut microbial composition remains relatively stable under the stress paradigm, but with the increase in disease severity, gut microbiota disturbances become inevitable. A similar explanation may apply to the present study, combined with a probably not large enough animal study group, resulting in statistically insignificant results.

Microbiome composition can be modified by oxidative stress within cells or tissues [48]. In diseases related to intestinal dysfunction associated with an improper diet, such as irritable bowel syndrome or obesity, stress is a significant risk factor affecting microbiome homeostasis, consequently causing dysbiosis [31]. Metagenomic analyses of the gut microbiome in mouse models after 16S rDNA sequencing revealed the influence of chronic stress on the modulation of the gut microbiome [31,36,46,48,49] and in models of stress associated with severe movement restriction [50,51].

### 2.7. DNA Damage and Repair

**Figure 9:**
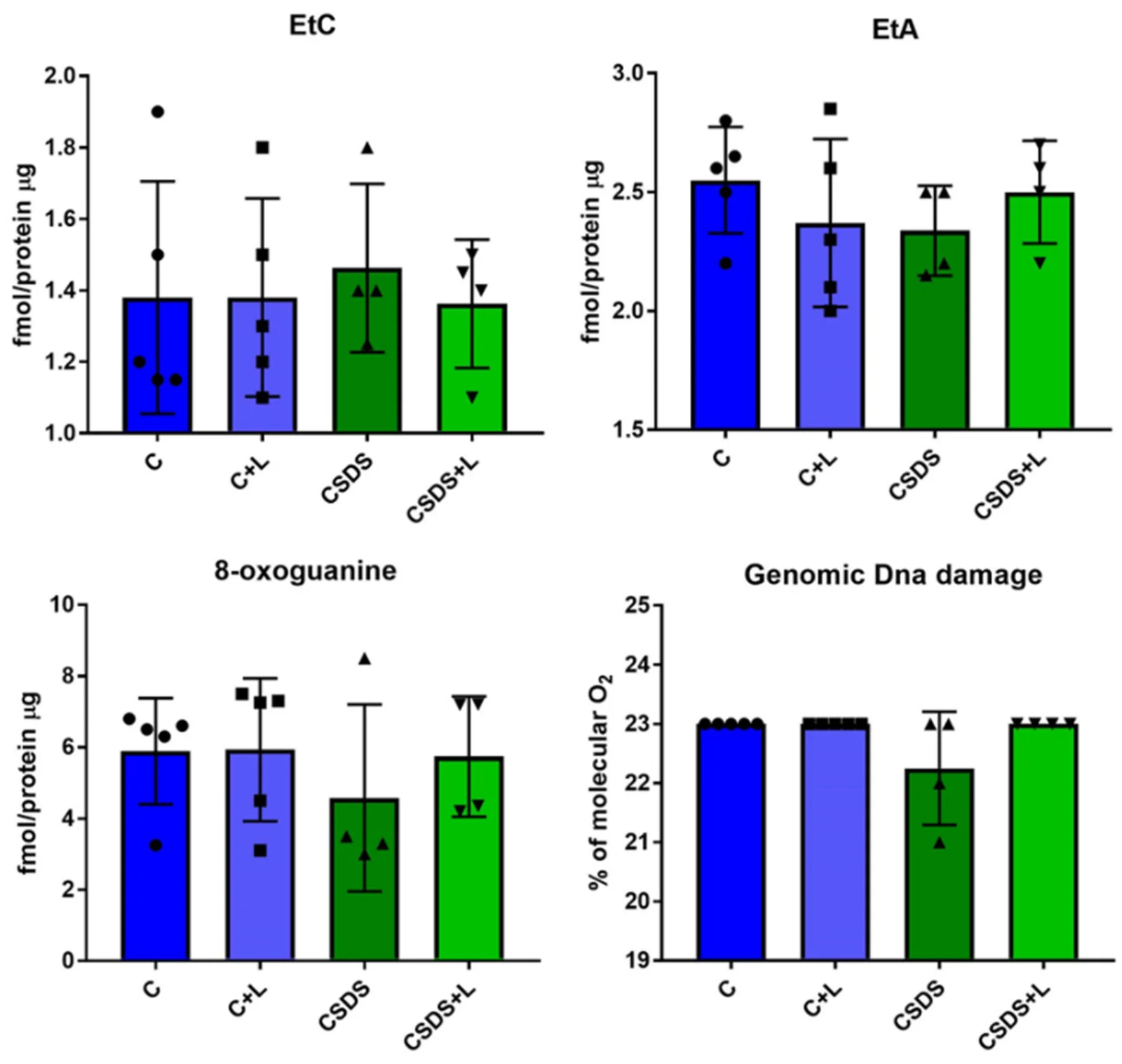
The effect of chronic social defeat stress (CSDS) and lactoferrin (L) administration on etheno-adducts εA, εC, and 8-oxo-G amounts measured in the mouse intestinal homogenates. Results are the mean ± SD; *n* = 4–5.

Analysis of DNA repair enzyme activity (nicking assay) for oxidative (8-oxoG) and etheno-adducts (εA, εC) revealed no significant differences between groups (Figure 9). The activity values corresponding to εC lesions were approximately two-fold lower than those detected for εA. Importantly, all εC values remained at levels comparable to the control group, indicating that L administration did not enhance this type of DNA modification. A similar outcome was observed for εA, and the modification levels were unchanged. In CSDS-affected samples, whether or not subjected to L, εA and εC values likewise showed no significant alterations relative to controls (Figure 9), whereas the highest trend in DNA damage was recorded for 8-oxoG. Here, the activity of repair enzymes was approximately twice that observed for εA and εC.

Oxidative stress is a known consequence of chronic social stress, leading to DNA base modifications. Interestingly, CSDS did not significantly alter the repair activities of ANPG, TDG, or OGG1 in intestinal homogenates. While 8-oxoG repair activity was generally higher than *etheno*-adduct repair, L administration did not modulate these levels. This suggests that the protective effects of L in this model are likely mediated through non-genomic pathways, such as direct antimicrobial activity or Tj stabilization, rather than by enhancing the DNA base excision repair capacity in the intestinal epithelium.

### 2.8. Study Limitation

Previous studies have demonstrated that L can exert biological activity at much lower doses (0.1–10 mg/kg), particularly in neurodevelopmental and neuroprotective contexts. Therefore, the present dose was selected to target a low-dose, physiologically relevant signaling range rather than a high-dose pharmacological, immunomodulatory effect [52,53]. The modest protective effects of L in our study may operate through multiple, non-mutually exclusive mechanisms. First, L possesses iron-sequestering properties that may selectively limit pathogenic bacterial growth while providing iron to beneficial commensals through specific uptake mechanisms [54]. Second, bioactive L-derived peptides (e.g., lactoferricins) possess antimicrobial and immunomodulatory activities that may contribute to microbiome stability and homeostasis under stress conditions [55]. Third, L receptors expressed on intestinal epithelial cells can activate intracellular signaling pathways involved in barrier integrity and stress responses [56].

The relatively small sample sizes for several endpoints limit statistical power and the generalizability of the findings. Therefore, the present study should be considered exploratory, and the observed effects require confirmation in larger, independently powered cohorts. A limitation is also the absence of a social interaction test to distinguish individual susceptibility and resilience to CSDS. All CSDS-exposed animals were analyzed as a single group, and potential differences in individual stress responsiveness may have influenced the observed group-level effects. The study was conducted exclusively in male mice, which may limit the generalizability of the findings, as CSDS paradigms in females typically require methodological adaptations. In addition, the 15-day stress protocol may not have been sufficient to induce the more pronounced dysbiosis observed in longer chronic stress paradigms. Furthermore, the absence of metabolomic analyses precluded the assessment of potential links between microbial alterations and specific functional metabolites, such as SCFAs. Therefore, additional studies are needed to further elucidate these observations.

## 3. Materials and Methods

### 3.1. Animals and Experimental Design

The chronic social defeat stress (CSDS) paradigm was performed in accordance with the standardized protocol for male mice described by Golden et al. (2011) [57]. C57BL/6J male mice (10–12 weeks old, wild-type, naïve to previous experimental procedures) were obtained from the outbred animal colony re-supplied by the Animal House of Mossakowski Medical Research Institute, Warsaw, Poland (Approval no. WAW2/149/2021, 2nd Local Ethics Committee for Animal Experimentation, Warsaw, Poland, as compliant with Polish Law) in accordance with Guidance on the operation of the Animals (Scientific Procedures) Act 1986 and associated guidelines, EU Directive 2010/63 for the protection of animals used for scientific purposes. Animals were maintained under standard specific pathogen-free (SPF) conditions on a 12 h light/dark cycle, with ad libitum access to food and water. A total of 69 mice were included in the study. Group sizes were: C (*n* = 17), C+L (*n* = 18), CSDS (*n* = 17), and CSDS+L (*n* = 17). The animals within each treatment group were divided between different experimental analyses. Exact sample sizes for each analysis are indicated in the corresponding figure legends. No animals were excluded based on experimental outcomes. One animal was excluded due to a technical complication during oral gavage. Data exclusion criteria were defined a priori, and no data points were removed except in cases of technical or measurement errors.

#### 3.1.1. Chronic Social Defeat Stress (CSDS) Protocol

The CSDS procedure was executed over 15 consecutive days. Male CD-1 mice were used as aggressors. C57BL/6J mice were exposed daily to a new, aggressive CD-1 mouse. Each C57BL/6J mouse was placed in the cage of a CD-1 mouse for 5 min, during which the CD-1 mouse exhibited physical aggression and hostile behavior. After the physical defeat phase, the C57BL/6J mouse was relocated to a section of a divided cage, separated by a perforated partition. This permitted sensory, but not physical, interaction between C57BL/6J and CD-1 aggressive mice for 24 h. A distinct CD-1 mouse was used for each daily defeat session to prevent habituation. Control mice were maintained under similar settings but were grouped with other C57BL/6J mice rather than with aggressor CD-1 animals. The mice were rotated daily in the partitioned cages to simulate sensory stress without suffering physical defeat. To minimize potential confounders, all groups were subjected to identical handling procedures throughout the experiment. Group allocation was known to the investigator performing the CSDS procedure and administering treatments; however, outcome assessment and statistical analyses were conducted in a blinded manner.

#### 3.1.2. Lactoferrin (L) Administration

Lactoferrin (bovine ≥ 85%, Merck, St. Louis, MO, USA, Cat. No. L1294) was freshly dissolved in sterile phosphate-buffered saline (PBS) and administered at a dose of 5 mg/kg body weight via oral gavage for 15 days. The dose of 5 mg/kg/day was selected based on previous preclinical studies demonstrating biological activity of orally administered bovine lactoferrin at this dose in mice [58]. Control animals received an equivalent volume (1 mL) of sterile PBS. Animals were randomly assigned to four groups: Control (C), Control + L (C+L), chronic social defeat stress (CSDS), and CSDS + L using a computer-generated randomization sequence.

### 3.2. Behavioral Tests

#### 3.2.1. Assessment of Fur Condition

The fur condition score provides a simple, non-invasive indicator of an animal’s overall health and welfare, can help detect general sickness, and may also offer indirect insight into the effects of L administration or stress, both of which can influence immune function, inflammation, and behavior. The condition of the fur was assessed every 2 days by two independent and blinded evaluators. The fur condition was evaluated based on a pre-defined scoring system for the whole body, head, back, and flanks. The scores ranged from 1 to 3 (half-point scores were also applied in the assessment), where 3 represented clean and well-groomed fur, 2 represented mildly unkempt fur, and 1 indicated matted or severely unkempt fur.

#### 3.2.2. Splash Test

The splash test was conducted to assess grooming behavior as an indicator of motivational or depressive-like states in mice. A 10% sucrose solution was prepared and sprayed onto the dorsal coat of each mouse using a spray bottle. Upon completion of the experimental procedure, the mouse was returned to its original housing cage. Mice were observed for 5 min, during which the total time spent grooming was recorded using a video camera. Grooming behavior, which included licking and cleaning the head, body, and tail, was scored by a blinded experimenter to reduce bias.

#### 3.2.3. Open Field Test

The open field test was used to measure general locomotor activity and anxiety-like behavior. The test was conducted in a square arena measuring 50 cm by 50 cm, with a 40 cm high wall. A central zone measuring 25 cm by 25 cm was defined within the arena. Each mouse was placed in the central zone of the open field and allowed to explore freely for 10 min. The arena was cleaned with 70% ethanol between trials to eliminate olfactory cues. An overhead camera recorded the mouse’s movement, and the total distance travelled and time spent in the peripheral zone were analyzed using tracking software such as EthoVision ver. XT7.

### 3.3. Physiological and Biochemical Analysis

#### 3.3.1. Body Weight Measurement

Body weight was recorded every 2 days. Changes were calculated as a percentage of baseline weight.

#### 3.3.2. Whole-Gut Transit

Mice were fasted for 6 h, then gavaged with 200 µL of 5% Evans Blue (EB) in 5% Arabic gum solution. Then, mice were provided with access to water, but food was withheld until the transit assessment was complete. The appearance of blue-colored faecal pellets was used as an indicator that the marker had transited through the gastrointestinal tract. The time elapsed between the oral administration of the EB solution and the first observation of blue faecal matter was recorded as the WGT, expressed in seconds. Upon completion of the assay, mice were returned to their standard feeding regimen. Throughout the procedure, animals were monitored for signs of distress or discomfort, and care was taken to ensure their well-being.

#### 3.3.3. Intestinal Permeability

Intestinal permeability was assessed by measuring plasma FITC-dextran following oral administration. Mice were fasted for 4 h with free access to water and received 150 µL of FITC-dextran (4 kDa; 80 mg/mL in PBS) by oral gavage (12 mg/mouse). At 1 and 2 h after administration, mice were euthanized by decapitation, and blood was collected directly into heparinized tubes. Approximately 500 µL of blood was collected per animal. Plasma was obtained by centrifugation at 3500× *g* for 5 min and stored at −80 °C until analysis.

Plasma samples were diluted 1:1 with PBS, and 100 µL was transferred to each well of a 96-well plate. The intensity of fluorescence was measured at 485 nm excitation and 528 nm emission. FITC-dextran concentrations were calculated from a standard curve ranging from 45 to 0.1758 ng/mL (two-fold serial dilutions); 0.1758 ng/mL was the lowest detectable standard above the blank. Plasma fluorescence was corrected for the 1:1 dilution factor. Assay sensitivity was defined by the lowest standard concentration reliably distinguishable from the blank (0.1758 ng/mL).

#### 3.3.4. Serum Biochemical Analysis

Concentrations of mouse serum glucose, total protein, amylase, lipase, and alkaline phosphatase (ALP) in Control, L, CSDS, and L+CSDS mice were analyzed using IDEXX Catalyst One (IDEXX Laboratories, Inc., Westbrook, ME, USA). Briefly, blood was immediately collected into a heparin tube, and according to IDEXX protocols, Chem 15 CLIP was used. Approximately 50–100 µL of the sample was loaded into the sample cartridge. For each analyte, the appropriate IDEXX slide specific to the parameter being tested was inserted into the device [59].

### 3.4. Tissue Analysis

#### 3.4.1. Histology & Immunohistochemistry

Jejunum and ileum sections were fixed in 4% paraformaldehyde at 4 °C for 15 min to preserve tissue structure. Following fixation, the tissue was washed with PBS to remove residual fixative. The cryoprotected tissue was embedded in OCT compound and then frozen using dry ice or liquid nitrogen. Tissue for immunohistochemistry was harvested from the small intestine, fixed in 4% paraformaldehyde at 4 °C overnight, and then cryoprotected in 30% sucrose in PBS. Tissues were embedded in OCT compound, and 15 µm-thick sections were prepared using a cryostat (Leica Microsystems, Wetzlar, Germany). Sections were mounted on glass slides (Superfrost Plus, Sigma-Aldrich, St. Louis, MO, USA, Cat. No. Z692255) and stored at −20 °C until further use. The following sections were blocked with 5% FBS in PBS containing 0.25% Triton X-100 for 1 h at room temperature, then incubated overnight at 4 °C with primary antibodies against ZO-1 (1:200, Invitrogen, Waltham, MA, USA, Cat. No. 61-7300) and claudin-5 (1:200, Thermo Fisher Scientific, Waltham, MA, USA, Cat. No. 35-2500) diluted in PBS containing 1% FBS. After a PBS wash, fluorescently labeled secondary antibodies (Alexa Fluor 488, Invitrogen, Waltham, MA, USA, Cat. No. A-11008) were applied for 1 h at room temperature in the dark. The nuclei were labeled with Hoechst 33258 (Sigma-Aldrich, St. Louis, MO, USA, Cat. No. B1155). Images were acquired in a confocal laser scanning microscope, LSM780 (Zeiss, Jena, Germany), and processed using the ZEN 2012 (Zeiss, Jena, Germany). Fluorescence intensities were measured using ImageJ software ver. 1.53T (NIH, Bethesda, MD, USA).

#### 3.4.2. Western Blot Analysis

Intestinal tissue was homogenized in Triton Lysis Buffer (20 mM Tris pH 6.8, 137 mM NaCl, 2 mM EDTA, 1% Triton X-100, 0.5 mM DTT, 1 mM PMSF), supplemented with Protease Inhibitor Cocktail and Phosphatase Inhibitor Cocktail (Protease Inhibitor: Sigma-Aldrich, St. Louis, MO, USA, Cat. No. P8340, Germany and Phosphatase Inhibitor Cocktail 2 (Sigma-Aldrich, St. Louis, MO, USA, Cat. No. P5726) and underwent centrifugation at 12,000× *g* for 20 min at 4 °C. Equal quantities of protein (20 µg) were separated on a 10% SDS-polyacrylamide gel and then transferred to a nitrocellulose membrane. Following overnight incubation with a ZO-1 antibody (1:400, Invitrogen, Waltham, MA, USA, Cat. No. 61-7300, predicted band size: ~230 kDa) or claudin 5 (1:500, Thermo Fisher Scientific, Waltham, MA, USA, Cat. No. 35-2500, predicted band size: ~23 kDa), the membranes were washed, treated with HRP-conjugated anti-rabbit IgG (1:2000; St. Louis, MO, USA, Cat. No. A0545), and subsequently developed Clarity Western ECL Substrate (Bio-Rad Laboratories, Hercules, CA, USA; Cat. No. 170-5061). GAPDH (Proteintech, Manchester, UK Cat. No. HRP-60004) served as the loading control.

For ZO-1, three biological replicates per group were analyzed in two technical replicates, and the mean densitometric value was used for statistical analysis. For claudin-5, four to five biological replicates per group were analyzed across two Western blot runs. Some biological samples were analyzed in both runs, whereas others were analyzed in a single run. The same control samples were included on both blots to ensure consistency between the two Western blot runs. Densitometric analysis was performed using ImageJ software. The intensity of each target protein band was normalized to the corresponding GAPDH band. An additional band at approximately 80 kDa was observed for ZO-1. Representative full-length Western blot images are provided in Supplementary File S1.

### 3.5. Microbiota Analysis

#### 3.5.1. Stool Sample Processing and DNA Extraction

Bacterial DNA was extracted from 60 to 70 mg of collected stool samples (day 1 and day 15) using Nucleospin DNA Stool Kit (Macherey-Nagel, Düren, Germany, Cat. No. 740472.50) according to the manufacturer’s protocol and suspended in 100 μL of elution buffer. Extraction efficiency was controlled via spike-in of Bacillus subtilis 16S (10^3^ copies/μL); mean recovery was 82.3 ± 4.1%.

#### 3.5.2. 16S rRNA Library Preparation and Sequencing

V3 and V4 regions of the 16S rRNA gene were amplified (550 bp product) using 5 ng/μl of isolated DNA, V3-V4 primers [60] and 0.5U KAPA HiFi HotStart ReadyMix PCR Kit (Roche Molecular Diagnostics, Basel, Switzerland). Then, 16S V3 and V4 amplicons were purified using a 0.8 ratio of AMPure XP beads (Beckman Coulter Life Sciences, Indianapolis, IN, USA). Sequencing libraries were dual-indexed (~630 bp product) using the Nextera XT Index kit (Illumina, San Diego, CA, USA) and purified using a 1.1 ratio of AMPure XP beads (Beckman Coulter Life Sciences, Indianapolis, IN, USA). The quality was determined on a Bioanalyzer DNA 1000 chip (Agilent Technologies, Santa Clara, CA, USA), and DNA concentration was measured using the Qubit High Sensitivity Kit (ThermoFisher, Waltham, MA, USA). All samples were normalized and sequenced (300 nt, paired-end reads) using the MiSeq Reagent Kit v3 (600-cycle) on the Illumina MiSeq platform.

#### 3.5.3. Bioinformatics and Statistics

For quality checking, NGS reads were subjected to FastQC software ver. v0.11.9 [61]. Raw paired-end sequencing reads were initially assessed for sequencing quality using FastQC software. The quality of the sequencing data was evaluated based on the Phred quality score, with reads meeting a quality threshold of >30 considered suitable for further analysis. Reads were trimmed with Trimmomatic [62] and then filtered with BBTools (based on their size) [63]. Remaining reads were analyzed using the QIIME (version 1.9.1) pipeline [64]. Thus, forward and reverse reads were merged using the fastq-join command [65], and operational taxonomic units (OTUs) were determined using an open-reference OTU picking process and the Greengenes database (threshold value of 97% sequence similarity). Chimera checking and removal were performed with ChimeraSlayer [66]. This workflow was based on established procedures for 16S rRNA gene amplicon sequencing and was performed in accordance with previously described methodology [67]. Normalization for diversity and statistical analysis was carried out using metagenomeSeq’s CSS (cumulative sum scaling) transformation [68].

Beta diversity and Principal Coordinates Analysis (PCoA) plots (unweighted and weighted UniFrac data) were generated using QIIME’s output and PhyloToAST software ver. v1.4.0 [69]. Additionally, a categorical variable analysis of similarities (ANOSIM) was performed. Microbial abundance was calculated and visualized with the phylo-seq package in R ver. 3.3.0 [70]. Alpha diversity metrics were analyzed with the Mann–Whitney U test, whereas taxonomic comparisons between groups were made using a nonparametric *t*-test provided by QIIME.

To predict putative metagenomic functional profiles, PICRUSt was applied [71] using QIIME’s closed-reference OTU picking output [x4] and the KEGG Orthology database [72]. As these functional profiles are inferred from 16S rRNA gene sequencing data, they were considered predictive and hypothesis-generating rather than direct measurements of microbial metabolic activity. Statistical analysis and data visualization were performed using the STAMP software package ver. 2.1.3 [73].

### 3.6. DNA Repair Enzyme Activity

Protein extraction: intestinal tissues were homogenized in 4 volumes of 50 mM Tris–HCl, pH 7.5, buffer containing 1 mM EDTA and a protease inhibitor cocktail (Sigma Aldrich, Germany). The cell debris was removed by centrifugation (7000× *g*, 4 °C, 15 min), and the supernatant, used as the source of DNA repair enzymes, was collected and stored in aliquots at −80 °C. Protein concentration was determined by the Bradford method [74]. Nicking assay: adduct excision activity was measured using an oligodeoxynucleotide duplex containing a single 8oxoG, ethenoA, and ethenoC residue according to the manufacturer’s instructions and previous analysis [75,76]. Briefly, the oligonucleotides (40-mers) containing at position 20 εA, εC, or 8-oxoG were labelled at the 5′ end with T4 polynucleotide kinase in the presence of 25µlCi [γ-^32^P] ATP (3000Ci/mmol) in the buffer prepared by the manufacturer. The samples were incubated at 37 °C for 1 h. Purification of the samples from unreacted ATP residues was performed using Micro Bio-Spin P-30 Tris chromatographic columns (Bio-Rad Laboratories). The samples were applied to the previously prepared columns and centrifuged at room temperature (8 min, 1000× *g*). The filtrate contained the purified oligonucleotides, and the unreacted ATP was retained in the column bed. The radiolabelled oligonucleotides were hybridized with complementary oligonucleotides containing the appropriate dT, dG, or dC against the modified base. The oligonucleotides were suspended in STE buffer and then mixed in a molar ratio of modified oligonucleotide: complementary oligonucleotide 1:2. Samples were incubated at 80 °C for 3 min and then cooled to room temperature for 2–3 h. The reaction mixture contained 5′^32^P duplexes (0.2 pmol), a reaction buffer, and incremental protein extracts (1 µg, 5 µg, 15 µg, 25 µg, 50 µg). The reaction was carried out for 1 h at 37 °C. Since repair enzymes and some other proteins have a strong affinity for DNA, which interferes with the analysis of the products, after the end of the enzymatic reaction, the protein of the tissue extract was digested with proteinase K (20 μg/sample) for 1 h at 37 °C. The 8-oxoG oligomer was further cut by incubation in 0.2 N NaOH at 70 °C for 15 min. After the reaction was complete, the Stop Solution with 95% formamide was added to the tests, and electrophoretic separation of the reaction products was carried out. The reaction products were separated using 20% polyacrylamide gels with 7 M urea as the denaturing agent. Samples were denatured by heating for 3 min at 90 °C, cooled on ice for app. 3 min. and briefly vortexed. The electrophoresis was carried out in a TBE buffer for 2–3 h at a voltage of 400–600 V (using a CONSORT 3000v-300mA high-voltage power supply). The gels were then autoradiographed for 12–16 h at −80 °C, and the resulting autoradiographs were scanned on a Personal Laser Densitometer SI (Molecular Dynamics, Sunnyvale, CA, USA) and analyzed using ImageQuant 5.2 and Microcal Origin programs. The percentage of cleavage of the known amount of ^32^P of the labeled oligonucleotide to the total was estimated and converted to product fmoles/hour/µg protein.

#### Real-Time PCR

Expression of 8-oxoguanine DNA glycosylase (OGG1), DNA-3-methyladenine glycosylase (ANPG), and thymine DNA glycosylase (TDG) was analyzed using SYBR Green chemistry. Data were normalized to 18S rRNA using the 2−ΔΔCT method [77]. Briefly, total RNA was extracted with the PureLink RNA kit (Invitrogen). RNA quantity and purity were determined using a NanoDrop microspectrophotometer ND-1000 (Wilmington, DE, USA). The concentration of RNA samples was determined by measuring optical density at 260 nm. One μg of total RNA was reverse-transcribed to high-capacity cDNA using a reverse transcription kit that included a mixture of random primers (Applied Biosystems, Foster City, CA, USA, Cat. No. 4368814). RT-PCR analysis was conducted by SYBR Green I dye detection (Applied Biosystems) using the real-time detection system (Bio-Rad). Primers were designed with PrimerExpress software ver. v3.0. (Table 1).

### 3.7. Statistical Analysis

Statistical analyses were performed using Prism 7 (GraphPad Software Inc., La Jolla, CA, USA). Results were expressed as mean ± standard deviation (SD), and the individual mouse was considered the experimental unit. The statistical approach was selected according to the experimental design and the characteristics of each response variable. For response variables to which the factorial design was applicable, the data were subsequently analyzed using two-way ANOVA, with CSDS exposure and lactoferrin administration as the two factors. The main effects of CSDS and lactoferrin, as well as their interaction, were evaluated. Where appropriate, multiple comparisons were performed using Šídák’s multiple-comparison test. For variables measured repeatedly in the same animals over time, an appropriate repeated-measures approach was applied. Microbiota-related analyses were evaluated separately according to the characteristics of the data. Alpha diversity was analyzed using the Mann–Whitney U test, beta diversity was assessed using PERMANOVA with 999 permutations (adonis2), and taxonomic comparisons were performed using the nonparametric test implemented in QIIME.

A *p*-value of less than 0.05 was considered statistically significant, with *** indicating *p* < 0.001, ** indicating *p* < 0.01, and * indicating *p* < 0.05. Sample sizes for the different experimental endpoints were based on previous studies using similar experimental paradigms and our prior experience, as no formal power calculations were performed for the individual endpoints. A summary of the response variables, sample sizes, experimental units, and statistical approaches used for each endpoint is provided in Table 2.

## 4. Conclusions

This study demonstrates that lactoferrin (L) administration attenuated specific somatic and anxiety-like behaviors induced in mice by chronic social defeat stress (CSDS), stabilizing fur condition and grooming latency in the splash test. While the open field test did not reveal significant differences in locomotion or stress-like features, the physical improvements (reduced body mass loss) suggest that L supports general vitality under stress. This aligns with the morphological findings in the ileum, where L partially mitigated stress-induced reduction in villi width and changes in crypt depth/number, suggesting a structural basis for improved nutrient absorption.

Collectively, our findings position L as a promising, gut-targeted nutritional adjunct that may serve as an attenuator of the somatic and intestinal consequences of chronic stress, even in the absence of large-scale alterations in microbiota composition. Future studies incorporating sex-specific models will determine the generalizability and translational relevance of these effects. These findings are exploratory and require confirmation in larger, adequately powered studies.

## Figures and Tables

**Table 1:** Oligonucleotide primers.

Gene	Primer	Sequence (5′ to 3′)
*OGG1*	*OGG1F*	CTCAGAAATTCCAAGGTGTTC
	*OGG1R*	CCGCTCCACCAT-GCCAGTG
*18S*	*18SpfF*	ATCCTTCGATGTCGGCTCTT
	*18SpfR*	ACTAACCTGTCTCACGACGGTC
*ANPG*	*ANPGF*	CGCAGCATCTATTTCTCAAGC
	*ANPGR*	GTGCCATTAGGAAGTCGCC
*TDG*	*TDGF*	TAATGGGCAGTGGATGACCC
	*TDGR*	TGCAGCATTTAAGCAGAGCTGA

**Table 2:** Summary of response variables, sample sizes, and statistical analyses.

Experimental Domain	Response Variable	Figure	*n*	Statistical Analysis
Physical assessment	Fur condition score	Figure 1A–B	9	Two-way repeated-measures ANOVA
Behavior	Grooming time—Splash test	Figure 1C	9	Two-way ANOVA
Behavior	Total distance—Open Field	Figure 1D	7	Two-way ANOVA
Behavior	Time in central zone—Open Field	Figure 1E	7	Two-way ANOVA
Body weight	Body weight over time	Figure 2A	5	Two-way repeated-measures ANOVA
Body weight	Body weight amplitude	Figure 2B	5	Two-way ANOVA
Serum biochemistry	Total protein	Figure 2C	6–9	Two-way ANOVA
Serum biochemistry	Glucose	Figure 2D	6–9	Two-way ANOVA
Serum biochemistry	Amylase	Figure 2E	6–9	Two-way ANOVA
Serum biochemistry	Lipase	Figure 2F	6–9	Two-way ANOVA
Serum biochemistry	ALP	Figure 2G	6–9	Two-way ANOVA
Intestinal permeability	Plasma FITC-dextran, 1 h	Figure 3A	4–5	Two-way ANOVA
Intestinal permeability	Plasma FITC-dextran, 2 h	Figure 3A	4–5	Two-way ANOVA
Gastrointestinal motility	Whole-gut transit time	Figure 3B	4–5	Two-way ANOVA
Western blot	ZO-1 expression	Figure 4A	3	Two-way ANOVA
Western Blot	Claudin-5 expression	Figure 4B	4–5	Two-way ANOVA
Immunohistochemistry	ZO-1 and Claudin-5 fluorescence intensity in jejunum	Figure 4C	4–5	Two-way ANOVA
Immunohistochemistry	ZO-1 and Claudin-5 fluorescence intensity in ileum	Figure 4D	4–5	Two-way ANOVA
Intestinal morphology	Jejunum	Figure 5A	3	Two-way ANOVA
Intestinal morphology	Ileum	Figure 5B	3	Two-way ANOVA
Microbiota diversity	Chao1	Figure 6A	3–4	Mann–Whitney U test
Microbiota diversity	Observed OTUs	Figure 6B	3–4	Mann–Whitney U test
Microbiota diversity	Faith’s PD	Figure 6C	3–4	Mann–Whitney U test
Microbiota diversity	Shannon index	Figure 6D	3–4	Mann–Whitney U test
Taxonomic composition	Relative abundance of major phyla	Figure 7	3–4	Taxonomic comparison using a nonparametric test in QIIME
Microbiota composition	Firmicutes/Bacteroidetes ratio	Figure 8	3–5	Repeated-measures analysis
DNA damage/repair	8-oxoG repair activity	Figure 9	4–5	Two-way ANOVA
DNA damage/repair	εA repair activity	Figure 9	4–5	Two-way ANOVA
DNA damage/repair	εC repair activity	Figure 9	4–5	Two-way ANOVA

## Data Availability

Data supporting reported results can be made available on request.

## Supplementary Materials

The following supporting information can be downloaded at https://www.mdpi.com/article/10.3390/molecules31203614/s1. Supplementary File S1, Representative raw Western blot images demonstrating protein band quality, with descriptions of the corresponding blots.

## Abbreviations

ALP, alkaline phosphatase; ANPG, DNA-3-methyladenine glycosylase; CSDS, chronic social defeat stress; EB, Evans blue; etA, 1, N6-ethenoadenine; etC, 3, N4-ethenocytosine; etG, N2,3-ethenoguanine or 1, N2-ethenoguanine (etheno-adducts); F/B, Firmicutes-to-Bacteroidetes ratio; FITC, fluorescein isothiocyanate-dextran; L, lactoferrin; LPS, lipopolysaccharide; OGG1, 8-oxoguanine DNA glycosylase; OTUs, operational taxonomic units; PBS, phosphate-buffered saline; PCoA, principal coordinates analysis; SCFAs, short-chain fatty acids; SD, standard deviation; TDG, thymine DNA glycosylase; TJ, tight junctions; WGT, whole gut transit.
