# Supplementary material for: Lactoferrin Modulates Intestinal Morphology and Microbiota Homeostasis in Chronic Social Defeat Stress Mice
Source: Molecules. 2026 Oct 10;31(20):3614. doi: 10.3390/molecules31203614 (PMC13707845; doi:10.3390/molecules31203614)

Representative raw Western blot images demonstrating protein band quality.

Claudin-5

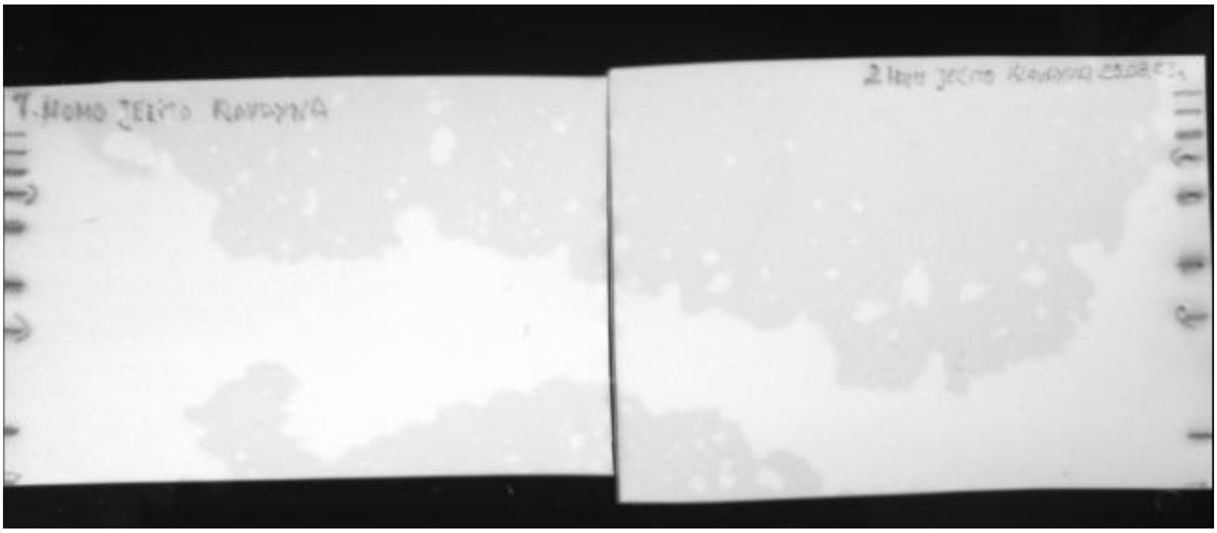

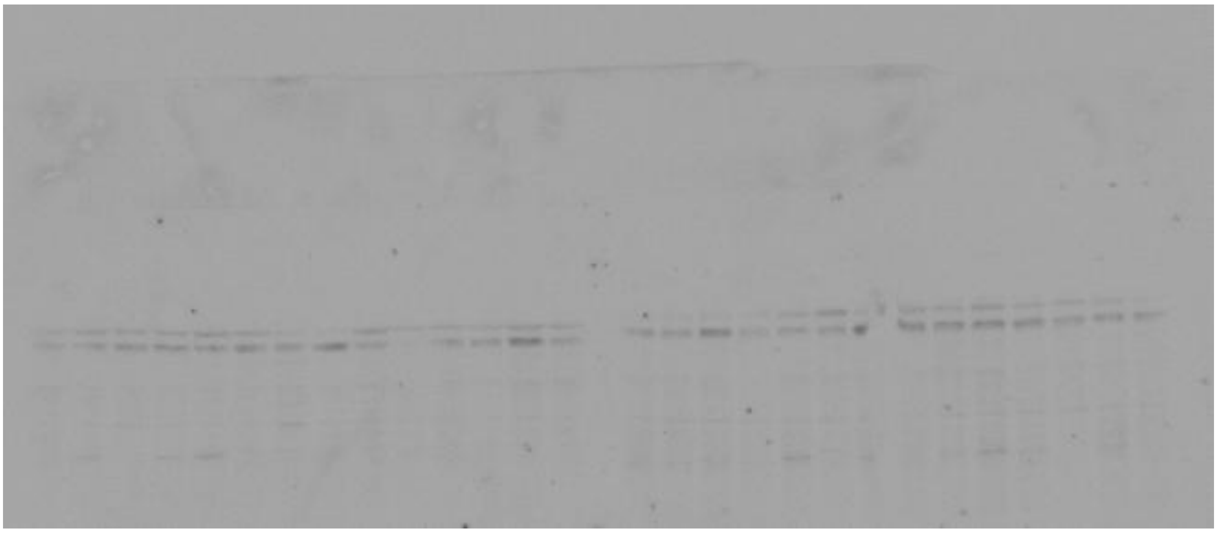

The upper panel shows the molecular weight marker, while the lower panel presents the chemiluminescent signal obtained after incubation with an anti–claudin-5 antibody (1:500, Thermo Fisher Scientific; predicted band size: ~23 kDa). Below, the corresponding GAPDH signal (~36 kDa) is shown, detected on the same membrane after stripping and used as a loading control.

The order of cortical homogenate samples, starting from the molecular weight marker, was as follows:

Left panel: four control samples (K11, K12, K15, K16), three control + lactoferrin samples (K5L, K6L, K13L), four samples from mice subjected to CSDS (K1D, K2D, K7D, K18D), and three samples from CSDS + lactoferrin mice (K3DL, K9DL, K10DL).

Right panel: four control samples (K11, K12, K15, K16), three control + lactoferrin samples (K5L, K14L, K20L), four samples from mice subjected to CSDS (K1D, K2D, K8D, K18D), and three samples from CSDS + lactoferrin mice (K9DL, K10DL, K17DL).

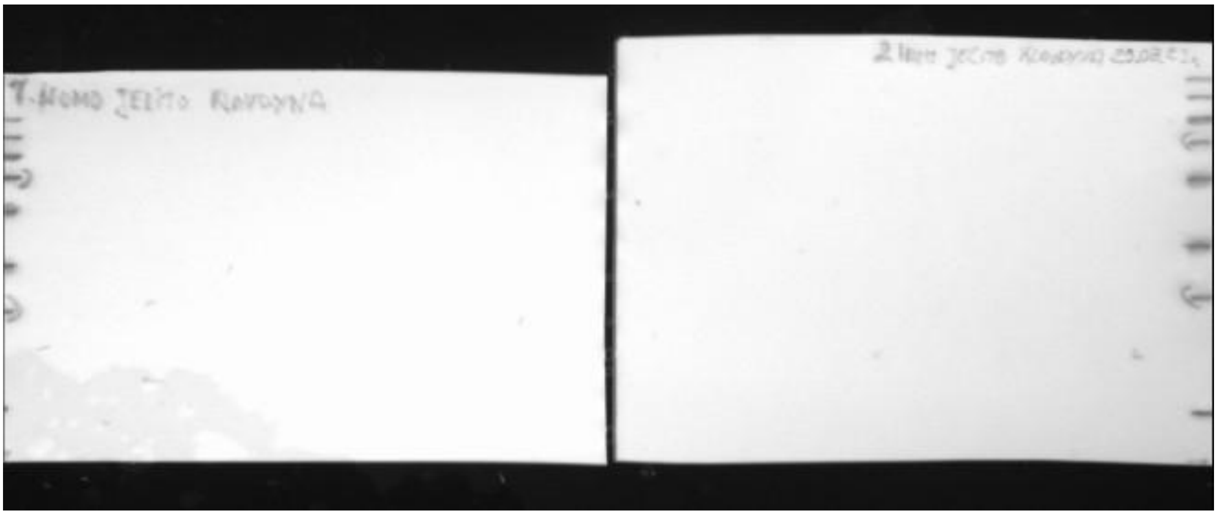

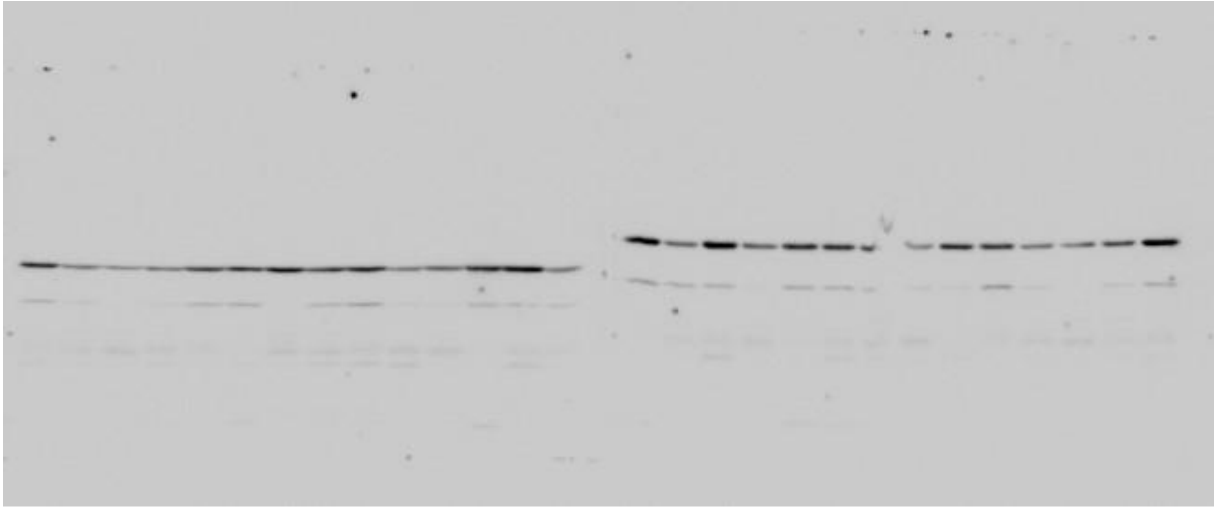

Zonula occludens-1

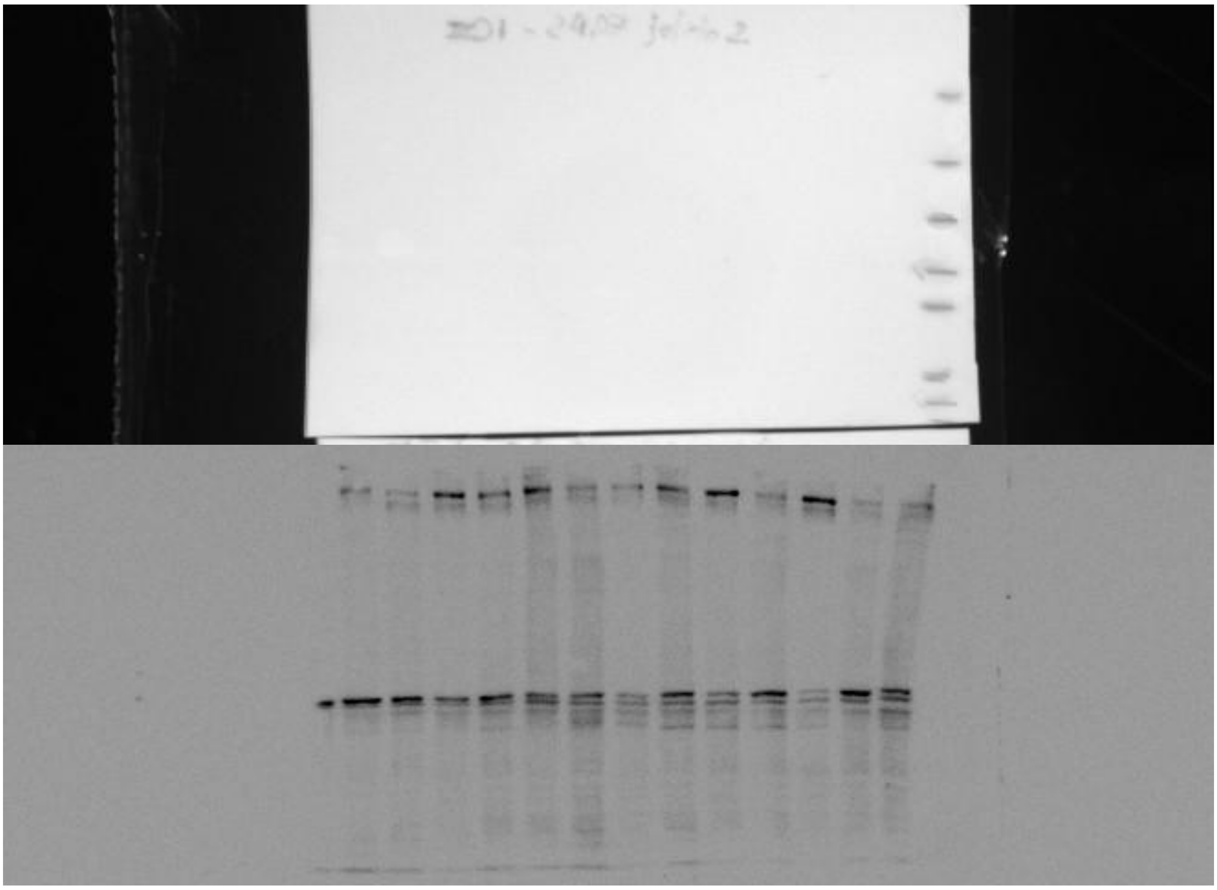

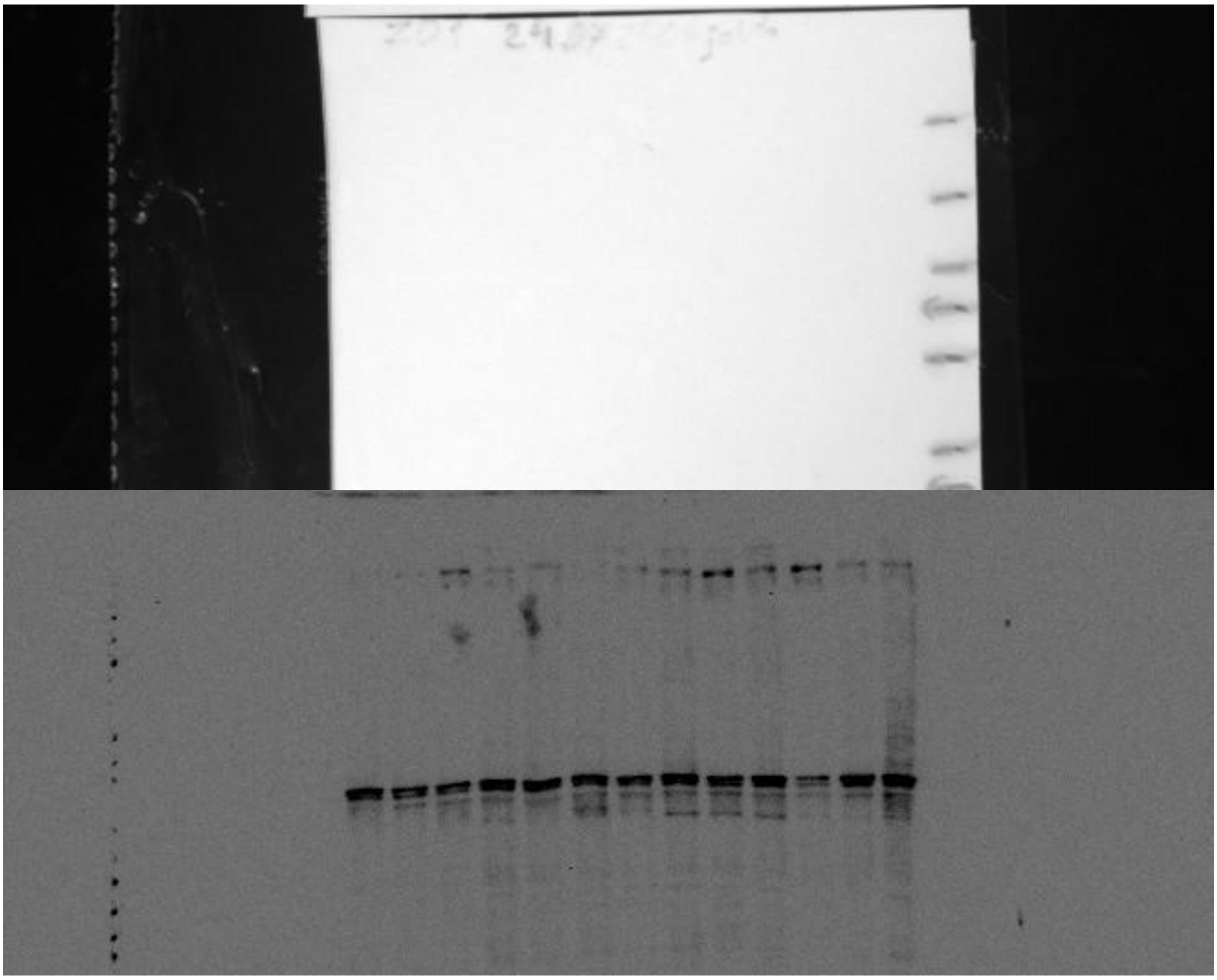

The upper panels show the molecular weight markers, while the lower panels present the chemiluminescent signal for ZO-1 obtained from two independent experimental sets after incubation with an anti–ZO-1 antibody (1:500, Thermo Fisher Scientific; predicted band size: ~230 kDa). An additional band at ~80 kDa was also observed. Below, the corresponding

GAPDH signal (~36 kDa) is shown, detected on the same membrane after stripping and used as a loading control.

The order of cortical homogenate samples, starting from the molecular weight marker, was as follows:

Upper panel: four control samples (K11, K12, K15, K16), three control + lactoferrin samples (K13L, K14L, K20L), four samples from mice subjected to CSDS (K1D, K2D, K8D, K18D), and three samples from CSDS + lactoferrin mice (K3DL, K9DL, K10DL).

Lower panel: four control samples (K11, K12, K15, K16), three control + lactoferrin samples (K13L, K14L, K20L), three samples from mice subjected to CSDS (K1D, K2D, K8D), and three samples from CSDS + lactoferrin mice (K3DL, K9DL, K10DL). The last two bands were not clearly detectable and were therefore excluded from the analysis.

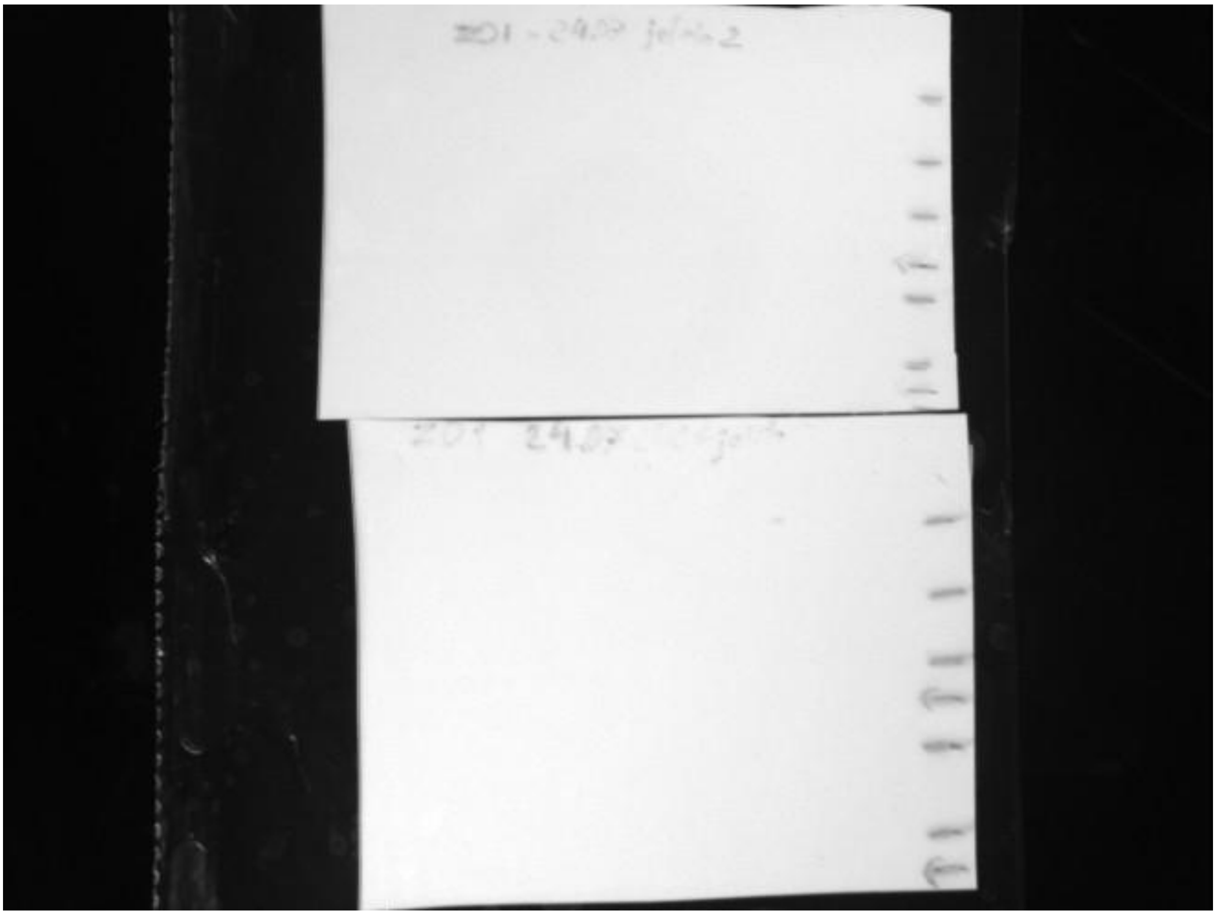

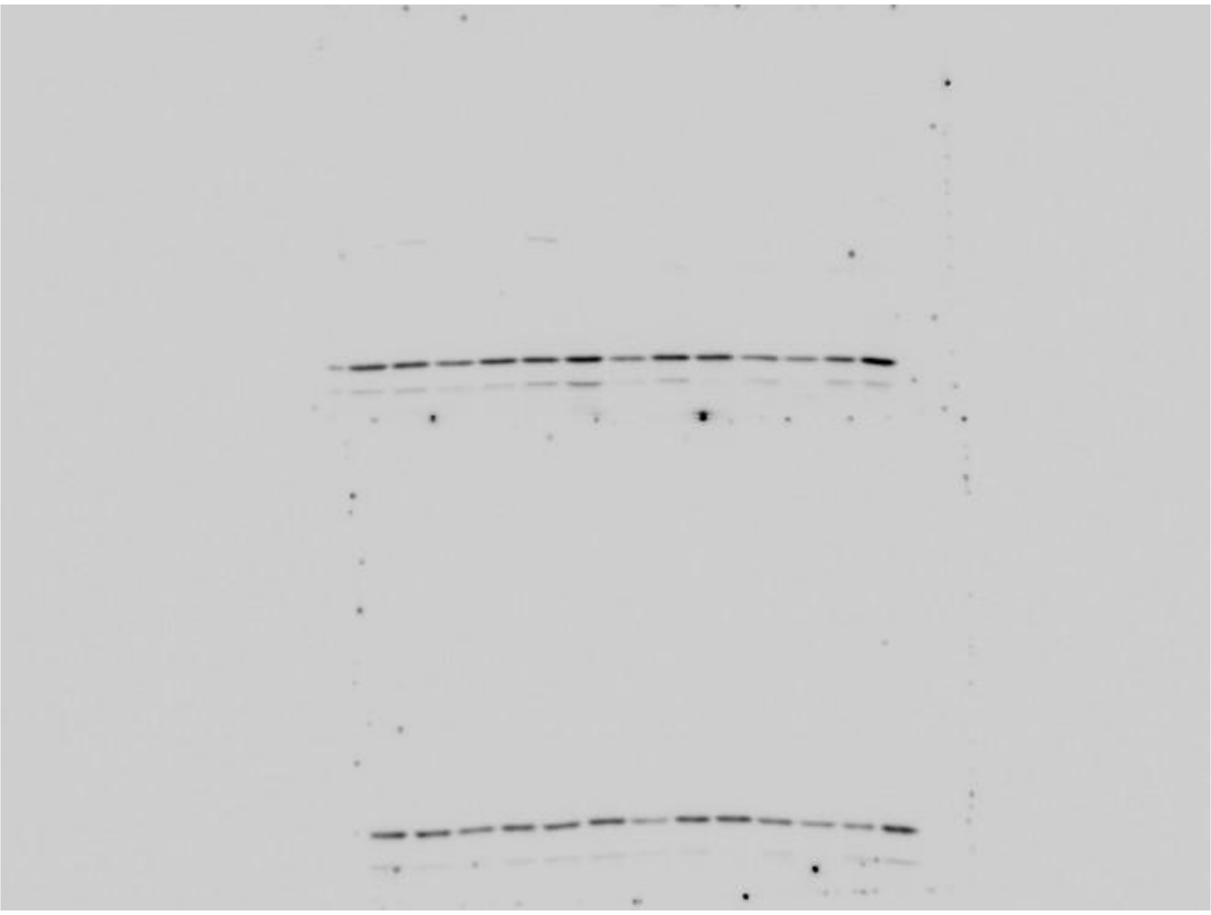

Supplement: Supplementary file 1 [file molecules-31-03614-s001.zip › molecules-4561066-supplementary.pdf]
